# Human lung epithelial BEAS-2B cells exhibit characteristics of mesenchymal stem cells

**DOI:** 10.1371/journal.pone.0227174

**Published:** 2020-01-03

**Authors:** Xiaoyan Han, Tao Na, Tingting Wu, Bao-Zhu Yuan

**Affiliations:** Cell Collection and Research Center, National Institutes for Food and Drug Control, Beijing, China; Università degli Studi della Campania, ITALY

## Abstract

BEAS-2B was originally established as an immortalized but non-tumorigenic epithelial cell line from human bronchial epithelium. Because of general recognition for its bronchial epithelial origin, the BEAS-2B cell line has been widely used as an *in vitro* cell model in a large variety of studies associated with respiratory diseases including lung carcinogenesis. However, very few studies have discussed non-epithelial features of BEAS-2B cells, especially the features associated with mesenchymal stem cells (MSCs), which represent a group of fibroblast-like cells with limited self-renewal and differentiation potential to various cell lineages. In this study, we compared BEAS-2B with a human umbilical cord-derived MSCs (hMSCs) cell line, hMSC1, which served as a representative of hMSCs in terms of expressing common features of hMSCs. It was observed that both BEAS-2B and hMSC1 shared the same expression profile of surface markers of hMSCs and exhibited similar osteogenic and adipogenic differentiation potential. In addition, like hMSC1, the BEAS-2B cell line exhibited suppressive activities on proliferation of mitogen-activated total T lymphocytes as well as Th1 lymphocytes, and IFNγ-induced expression of IDO1, all thus demonstrating that BEAS-2B cells exhibited an almost identical characteristic profile with hMSCs, even though, there was a clear difference between BEAS-2B and hMSCs in the effects on type 2 macrophage polarization. Most importantly, the hMSCs features of BEAS-2B were unlikely a consequence of epithelial-mesenchymal transition. Therefore, this study provided a set of evidence to provoke reconsideration of epithelial origin of BEAS-2B.

## Introduction

The BEAS-2B cell line has been a widely used immortalized but non-tumorigenic human cell line established from normal human bronchial epithelium obtained from a non-cancerous individual by Curtis C. Harris’ group in 1988 [1]. The cell line was established via transfection with an adenovirus 12-SV40 hybrid virus and subsequent immortalization via consecutive cell passaging [1]. Since being labeled as a bronchial epithelial cell line, BEAS-2B has been extensively used to study cellular and molecular mechanisms involved in lung carcinogenesis, including the role of epithelial-mesenchymal transition (EMT) in lung carcinogenesis [2–4], as well as pneumococcal infections [5]. In addition, the BEAS-2B cell line has been utilized as an *in vitro* cell model for assaying or screening various chemicals and biological agents with potential pulmonary toxicity or lung carcinogenicity [6–8]. While very few of these studies provided further evidence regarding the expression of proteins, such as vimentin, cytokeratin 8 and E-cadherin [9], to support epithelial essence of BEAS-2B, the vast majority of the studies did not even present concern about the epithelial features of BEAS-2B. However, as a widely used cell line, any further characterization regarding its epithelial origin will help clarify or validate the findings achieved from using this cell line, or help develop it as a valuable experimental tool in new studies.

Mesenchymal stem cells (MSCs) are fibroblast-like stem cells existing in almost all tissues, such as bone marrow, umbilical cord, adipose tissue, dental pulp, etc. [10–13]. They have substantial self-renewal and differentiation potential [14, 15]. Currently, human MSCs (hMSCs) of different tissue origins are commonly defined following a minimum criteria, which are in plastic-adherent growth; expressing CD90, CD105, and CD73 surface markers in over 95% cell populations and CD45, CD34, CD14 or CD11b, CD19, and HLA-DR surface markers in less than 2% populations; being able to differentiate at least into osteocytes, adipocytes, and chondrocytes under each differentiation protocol [16–18]. In addition to these minimum criteria, hMSCs also exhibit unique immunomodulatory activities, including the inhibition of proliferation/activation of total T cell population as well as proinflammatory T cell subsets, such as Th1 or Th17 CD4^+^-T lymphocytes, and the promotion of proliferation/polarization of regulatory T lymphocytes (Tregs) and type 2 macrophages in both *in vitro* and *in vivo* assays [19–21]. All these immunomodulatory activities are mediated in part by the molecules secreted by hMSCs, such as indoleamine 2, 3-dioxygenase 1 (IDO1) and prostaglandin E_2_ (PGE_2_) [22, 23]. Because of the unique immunomodulatory activities and differentiation potential, hMSCs of different tissue origins have been used as the most popular type of stem cells in clinical studies for treating various diseases, including graft-versus-host disease (GvHD), liver fibrosis, stroke, multiple sclerosis and systemic lupus erythematosus [24, 25].

Encouraged by our unintentional observations revealing that BEAS-2B cells expressed a set of definitive surface markers of hMSCs, we performed in this study a more comprehensive characterization for BEAS-2B in comparison with hMSCs, including the characterizations for surface markers of hMSCs, differentiation potential and immunomodulatory activities. As a consequence, we revealed that, like hMSCs, the BEAS-2B cells expressed CD73, CD90, CD44, and CD105 without expressing CD45, CD34, CD14 or CD11b, CD19, and HLA-DR. In addition, the BEAS-2B cell line exhibited both osteogenic and adipogenic differentiation potential, and effectively inhibited proliferation of PHA-activated total T lymphocytes and Th1 lymphocytes from peripheral blood mononuclear cells (PBMCs).

Thus, our study provided comprehensive evidence to demonstrate for the first time that the BEAS-2B cell line shared several key characteristics with hMSCs. While raising concerns about the epithelial origin of the cell line, the study in fact helped improve our understanding on biological features of BEAS-2B cells, especially its non-epithelial features. More importantly, due to the possession of features of hMSCs, our study may support the use of BEAS-2B in other fields, such as using it as a reference cell line in the quality control of hMSCs.

## Materials and methods

### 1. Materials

#### Cells

The cell lines used in this study are the BEAS-2B, a human bronchial epithelial cell line, WI-38, a diploid fibroblast cell line of human fetal lung tissue origin, A549 and NCI-H1703, two human non-small cell lung carcinoma (NSCLC) cell lines, all of which were collected in our laboratory. PBMCs were collected from healthy donors in Beijing Red Cross center (37# of North 3rd Ring Road, Beijing). hMSC1, a human umbilical cord-derived mesenchymal stem cell line, with the catalog number of 20120822C6P5 was gifted anonymously from TuoHua Biotech company (Siping, China). The BEAS-2B, WI-38, A549, NCI-H1703, and hMSC1 cells were all cultured in alpha-MEM complete medium containing 10% FBS, 100 U/ml penicillin-streptomycin.

#### Chemicals

Human recombinant IFNγ and TGF-β1 proteins were from R&D Systems (Minneapolis, MN); phorbol 12-myristate 13-acetate (PMA), phytohemagglutinin (PHA), RepSox, a small molecule inhibitor of TGF-β1 signaling, and carboxyfluorescein diacetate succinimidyl ester (CFSE) were from Sigma–Aldrich (St. Louis, MI); Ionomycin and Brefeldin A were from Cell Signaling Technology (Danvers, MA).

#### Antibodies

The antibodies against IDO1, cytokeratin 8 (CK8), cytokeratin 18 (CK18), fibronectin, and SV40 Large T antigen were from Abcam (Cambridge, UK). The β-actin antibody and the epithelial cell adhesion molecule (EpCAM) antibody were from Sigma–Aldrich (St. Louis, MI). The E-Cadherin and Vimentin antibodies were from Cell Signaling Technology (Danvers, MA). The twist antibody was from Santa Cruz Biotechnology (Santa Cruz, CA); The fluorescin-conjugated antibodies for detecting CD8 (FITC), IFNγ (PE), CD3 (APC), CD14 (FITC), and CD206 (APC) were from BD Biosciences (San Diego, CA).

### 2. The nude mice tumorigenicity assay

Nude mice were purchased from the Laboratory Animal Center of National Institutes for Food and Drug Control (NIFDC) (Beijing, China). Each of 3- or 4-week old female BALB/c nude mice was injected with 2×10^6^ cells subcutaneously in the middle of dorsum of upper limb. The mice were examined for tumor growth twice a week after inoculation for at least three months. The animal use protocols for this assay were approved by the NIFDC Institutional Animal Care and Use Committee.

### 3. The flow cytometry assay for detecting cell surface markers

BD Stemflow hMSC Analysis Kit was employed to detect expression of cell surface markers using FACS Calibur flow cytometer (BD Biosciences) following the procedures reported in a previous study [26]. The data was collected and analyzed by FCS Express V3 software.

### 4. Short tandem repeat (STR) analysis

Genomic DNA of BEAS-2B was extracted by using Dnaeasy Blood & Tissue Kit (Qiagen), and then amplified by direct PCR for examining the expression of 9 STR loci, i.e. the loci of Amelogenin, CSF1PO, D13S317, D16S539, D5S818, D7S820, THO1, TPOX, vWA. The PCR products were separated and detected via capillary electrophoresis using ABI Prism 3130 followed by data analysis with GeneMarker software. The profile of PCR products was then used to validate cell identity and determine existence of cross-contamination between different cell lines.

### 5. Western blotting analysis

The procedures for conventional Western blotting were followed to monitor changes in expression of relevant proteins in different cell lines with or without treatments. Briefly, cell lysates were prepared using RIPA buffer containing proteinase inhibitor cocktail (Sigma), separated in 8–12% PAGE gel and transferred onto nitrocellulose membrane. The signals were detected using ECL Advance Western Blotting Detection Kit (GE Healthcare, Piscataway, NJ).

### 6. Osteogenic and adipogenic differentiation

The abilities of hMSC1 or BEAS-2B to differentiate into adipocytes and osteocytes were tested by using STEMPRO Differentiation Kit following the procedures described in a previous report [26]. Briefly, the cells growing in approximately 80% confluence were incubated at 37°C, 5% CO_2_ in each well of a 24-well cell culture plate with each differentiation induction medium for 21 days, then fixed with 4% formaldehyde for 30 min and stained with 0.3% Oil Red O solution for 50 min for testing adipogenesis or with 2% Alizarin Red S solution for 5 min for testing osteogenesis. After thorough washing with water, the images from each staining were taken under a light microscope. In each differentiation assay, the cells growing in each regular complete medium were used as the negative control.

### 7. Inhibition of Th1 lymphocyte proliferation by hMSC1 or BEAS-2B cells

Fresh PBMCs were co-cultured with hMSC1 or BEAS-2B cells at 1:5 ratio in RPMI 1640 complete medium for 18 hours and then stimulated with 25 ng/mL PMA, 1 μg/mL Ionomycin, and 10 μg/mL Brefeldin A together for another 5 h. Then, the CD3^+^CD8^−^IFN-γ^+^ cells were gated using BD-Calibur flow cytometer with relevant antibodies to determine the amount of Th1 lymphocytes [27]. The data was analyzed by FCS Express V3 software.

### 8. CFSE staining-based lymphocyte proliferation assay

For testing lymphocyte proliferation, the procedures reported in a previous study were followed [26]. Briefly, 1×10^6^ PBMCs isolated freshly using Ficoll solution and suspended in 1 ml PBS containing 5% FBS were incubated with 5 μM CFSE at room temperature for 5 min. After thorough washing with PBS containing 5% FBS, the CFSE-labeled PBMCs were then co-cultured with BEAS-2B cells or hMSC1 in 5:1 ratio for 7 days at 37°C and 5% CO_2_ in each well of a 12-well cell culture plate in RPMI-1640 complete medium containing 10 μg/ml PHA. After incubation, all lymphocytes were collected and the inhibition of lymphocyte proliferation was determined by gradual reduction of CFSE signal over the incubation period as detected by BD-Calibur flow cytometer. The data was further analyzed by FCS Express V3 software.

### 9. Induction of type 2 macrophage polarization

Fresh PBMCs were used for isolating CD14^+^ monocytes, which were sorted by anti-CD14 MicroBeads (Miltenyi). The CD14^+^ monocytes with over 90% purity were plated in 6-well cell culture plates with 0.5–1×10^6^ cells per well, and co-incubated with 2×10^5^ hMSC1 or BEAS-2B cells for 3–4 days. After staining with FITC-conjugated CD14 and APC-conjugated CD206, the macrophages were then tested for the expression of CD206 by flow cytometry. The data was collected and analyzed with FCS Express V3 software.

### 10. Quantitative RT-PCR

The quantitative RT-PCR was employed to detect mRNA expression of Slug and Snail in BEAS-2B cells after treatment with RepSox or TGF-β1 [28]. Briefly, 1 μg of total RNA isolated by Trizol reagent (Invitrogen) from the cells were reverse-transcribed by using the SuperScript III First-Strand Synthesis System (Invitrogen). The qRT-PCR assay was performed by using SYBR Premix Ex Taq II kit (Takara, Dalian, China) and the LightCycler 96 Real-time PCR system (Roche). The levels of Slug and Snail mRNAs were normalized to the level of GAPDH mRNA. The primers used for examining the expression of Slug, Snail and GAPDH were used: Slug, CCAAACTACAGCGAACTGGA and GTGGTATGACAGGCATGGAG; Snail, CTCTTTCCTCGTCAGGAAGC and GGCTGCTGGAAGGTAAACTC; GAPDH, GGTCTCCTCTGACTTCAACA and GTGAGGGTCTCTCTCTTCCT.

### 11. Statistical analysis

The data collected from flow cytometry were expressed as means±SEM of at least three separate experiments. The comparison between group means was assessed using one-way analysis of variance with Newman-Keuls posttest in GraphPad Prism 6 Software (San Diego, CA). The difference with p<0.05 was considered statistically significant.

## Results

### 1. BEAS-2B was validated as an immortalized non-tumorigenic human cell line expressing SV40 Large T antigen

**Fig 1:**
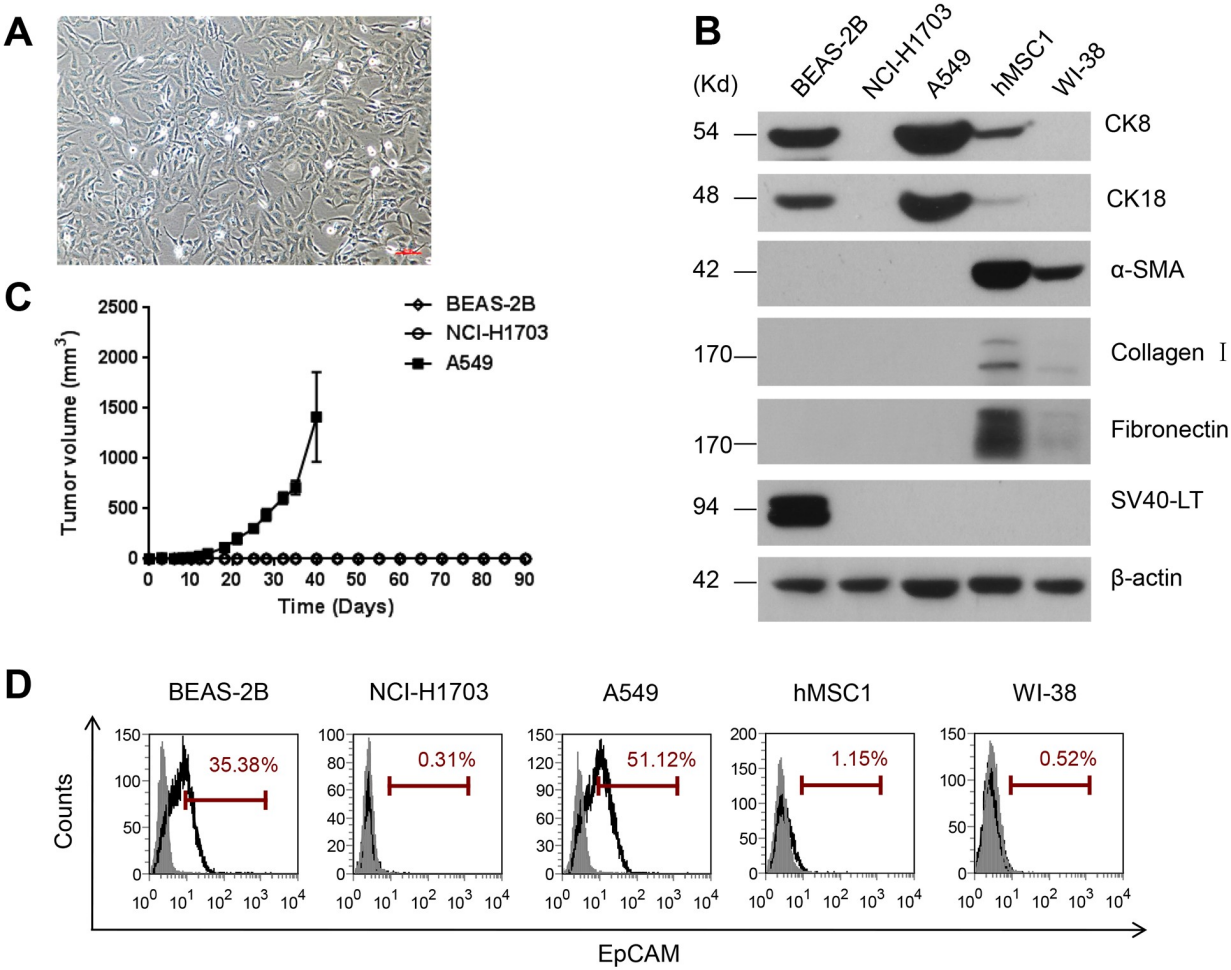
Validation of biological characteristics of BEAS-2B cells. (A). Contrast microscopy shows fibroblast-like morphology of BEAS-2B cells. The magnification is in 100 multiplication. Scale bar = 100 μm. (B). The expression of CK8, CK18, α-SMA, Collagen I, fibronectin, E-cadherin and SV40-LT proteins were examined by Western blotting in BEAS-2B, hMSC1, WI-38, NCI-H1703 and A549 cells, in which the expression of β-actin served as the loading control. (C). Nude mice tumor formation assay revealed that A549 represented a tumorigenic cell line with fast tumor formation in nude mice, while BEAS-2B and NCI-H1703 cells were non-tumorigenic with no detectable tumor formation in nude mice. (D). The EpCAM expression on BEAS-2B, NCI-H1703, A549, hMSC1, and WI-38 cells was detected by flow cytometry and the positivities of the expression in these cells were 35.38%, 0.31%, 51.12%, 1.15% and 0.52%, respectively.

The BEAS-2B cell line was established originally via immortalization of human bronchial cells using adenovirus12-SV40 hybrid virus [1]. In the original report, after characterizing the expression of SV40 Large T antigen (SV40-LT) and CK8 and CK18, both of which were assumed to be the epithelial proteins, the cell line was then accepted as a non-tumorigenic lung epithelial cell line. However, our characterizations revealed the BEAS-2B cells exhibiting characteristics of both epithelial and mesenchymal cells in both cell morphology and expression of marker proteins (Figs 1 and 3). In our characterizations, two mesenchymal cell lines, hMSC1 and WI-38, and two lung epithelial cancer cell lines, NCI-H1703 and A549, were included. Consistent to the original report, BEAS-2B was validated as a SV40-LT expressing cell line without tumorigenicity when inoculated in nude mice (Fig 1B and 1C).

However, in the test of marker proteins, it was found in Western blotting that the BEAS-2B cells were positive for CK8 and CK18 expression, but negative for α-SMA, collagen I and fibronectin, which are three proteins abundantly expressed in hMSCs. In comparison, the A549 cells were also positive for CK8 and CK18 while NCI-H1703 cells were negative for these two proteins. In addition, while the WI-38 cells were negative for CK8 and CK18, moderately positive for α-SMA but slightly positive for collagen I and fibronectin, the hMSC1 cells were positive for all marker proteins (Fig 1B). In addition, we also detected the expression of Epithelial Cell Adhesion Molecule (EpCAM) in BEAS-2B and A549 cells with the positivies being 35.38% and 51.12%, respectively. However, the EpCAM expression was hardly detectable in NCI-H1703, hMSC1 and WI-38 cells (Fig 1D). Putting all together, the new characterizations validated the original findings that BEAS-2B represented a non-tumorigenic cell line expressing SV40-LT, CK8, CK18, and EpCAM as well. However, it failed to support a conclusion that BEAS-2B was absolutely of epithelial origin as the expression of CK8, CK18 and even EpCAM were found in both epithelial and mesenchymal cells.

### 2. The BEAS-2B cell line used in our studies shared an identical genetic fingerprint with the same cell line deposited in ATCC

**Fig 2:**
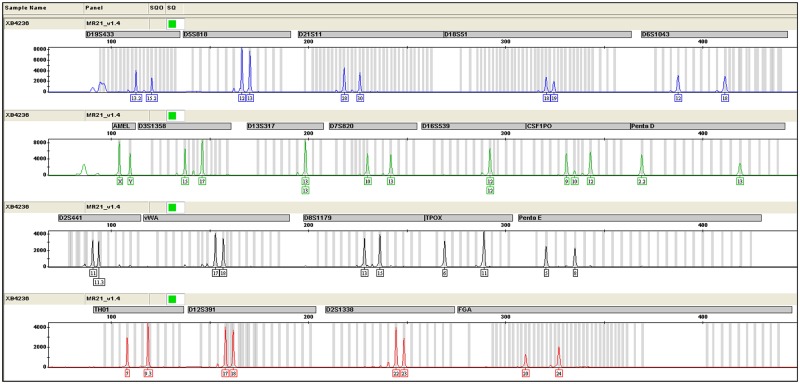
Histograms of a 9-loci STR panel for the BEAS-2B cells used in the present studies. The histograms was generated for the BEAS-2B cells used in the present studies via capillary electrophoresis to reveal a 9-loci STR profile covering the loci of Amelogenin, CSF1PO, D13S317, D16S539, D5S818, D7S820, THO1, TPOX and vWA.

To assure the BEAS-2B cell line used in our studies was identical with or originated from the same cell line deposited in American Tissue Collection Center (ATCC), we employed a short tandem repeat (STR) assay to examine genetic fingerprint of BEAS-2B using a 9-loci STR panel covering the loci of Amelogenin, CSF1PO, D13S317, D16S539, D5S818, D7S820, THO1, TPOX, and vWA (Fig 2), the same loci also employed by ATCC when reporting the identity of the same cell line. Through an online STR matching analysis using DSMZ profile database from www.dsmz.de/fp/cgi-bin/str.html, an evaluation value (EV) of 97% was obtained from the comparison between the BEAS-2B line deposited in ATCC and the line of ours (Table 1). As the two cell lines can be considered identical if the EV greater than 90%, we thus confirmed the BEAS-2B cell line used in our studies was identical to the cell line deposited in ATCC.

**Table 1:** Comparison for the STR profile between the BEAS-2B cell line used in the present studies and the cell line of the same name deposited in ATCC.

STR loci	ATCC	Our lab
Amelogenin	X,Y	X,Y
CSF1PO	9, 12	9, 10, 12
D13S317	13	13
D16S539	12	12
D5S818	12,13	12,13
D7S820	10,13	10,13
THO1	7, 9.3	7, 9.3
TPOX	6, 11	6, 11
vWA	17, 18	17, 18

The repeat numbers of 9 STR loci generated from the histograms for the BEAS-2B cell line used in the present studies are almost identical to the numbers of the same STR loci for the BEAS-2B cell line deposited in ATCC.

### 3. The BEAS-2B cell line shared an identical profile in cell surface markers with hMSCs

**Fig 3:**
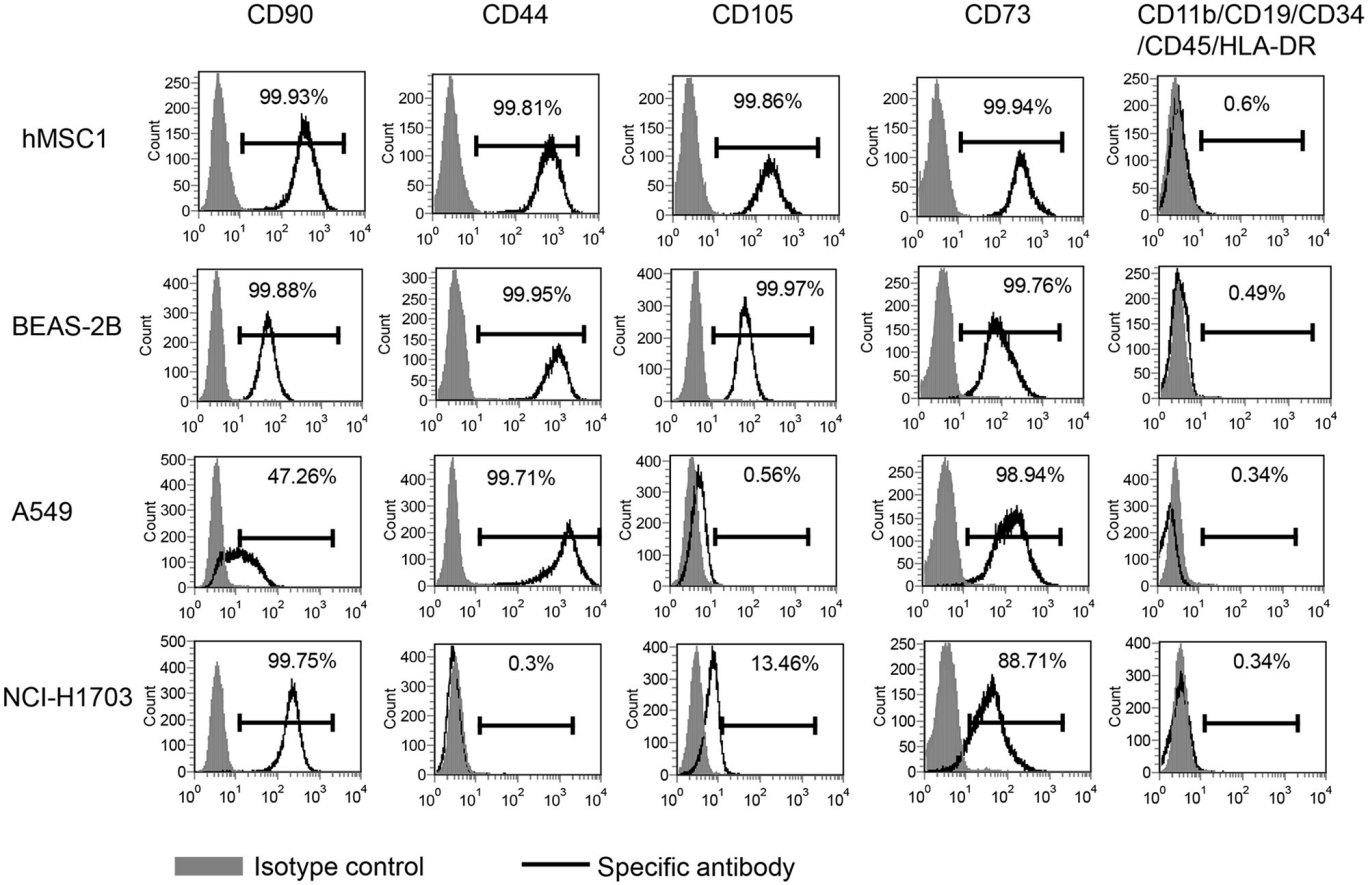
The BEAS-2B cells express a group of classical hMSCs surface markers. Flow cytometry assay detected the expression of surface markers CD90, CD44, CD105, CD73 and CD11b, CD19, CD34, CD45, HLA-DR in BEAS-2B, A549 and NCI-H1703 cells in comparison with hMSC1. Both hMSC1 and BEAS-2B showed positive staining in over 95% of cell population for CD90, CD44, CD105, and CD73. Meanwhile, the positive staining for CD90, CD44, CD105, and CD73 was found in 47%, 100%, 0.56% and 99% of A549 cells, respectively, and in 100%, 0.3%, 13% and 89% of NCI-H1703 cells, respectively. All four cell lines exhibited less than 1% positive staining for surface markers CD11b, CD19, CD34, CD45 and HLA-DR.

The BEAS-2B cell line has been frequently used as an *in vitro* non-tumorigenic lung epithelial model in a large variety of studies associated mostly with lung carcinogenesis with a few of them using it in epithelial-mesenchymal transition (EMT) studies [9]. However, no attempts in all the studies, even in EMT studies, have been made to further characterize the cell line for expressing the definitive mesenchymal surface markers. In this study, a group of positive and negative surface markers commonly used to define hMSCs was utilized to characterize BEAS-2B cells. It was then observed via flow cytometry assay that the BEAS-2B cell line shared an identical surface marker profile with hMSCs as both BEAS-2B and hMSC1 expressed positive surface markers CD44, CD73, CD90 and CD105 with over 99% positivity, but almost did not express negative surface markers CD11b, CD19, CD34, CD45 and HLA-DR with less than 1% positivity (Fig 3). Meanwhile, we examined these surface markers on both A549 and NCI-H1703 cells as well. It was found that the two epithelial cell lines almost did not express CD11b, CD19, CD34, CD45 and HLA-DR as well. However, even though over 95% of the A549 cells expressed CD44 and CD73, but less than 50% of the cells were positive for CD90 and less than 2% positive for CD105 (Fig 3). In NCI-H1703 cells, the expression for CD90, CD73, CD105 and CD44 were 99.75%, 88.71%, 13.46%, and 0.3%, respectively (Fig 3). Since all the surface markers chosen in this test were often used together to define mesenchymal stem cells, it was then strongly suggested that the surface marker profile of BEAS-2B appeared more like mesenchymal stem cells rather than epithelial cells.

### 4. The BEAS-2B cells shared a similar potential with hMSCs on both osteogenic and adipogenic differentiation

**Fig 4:**
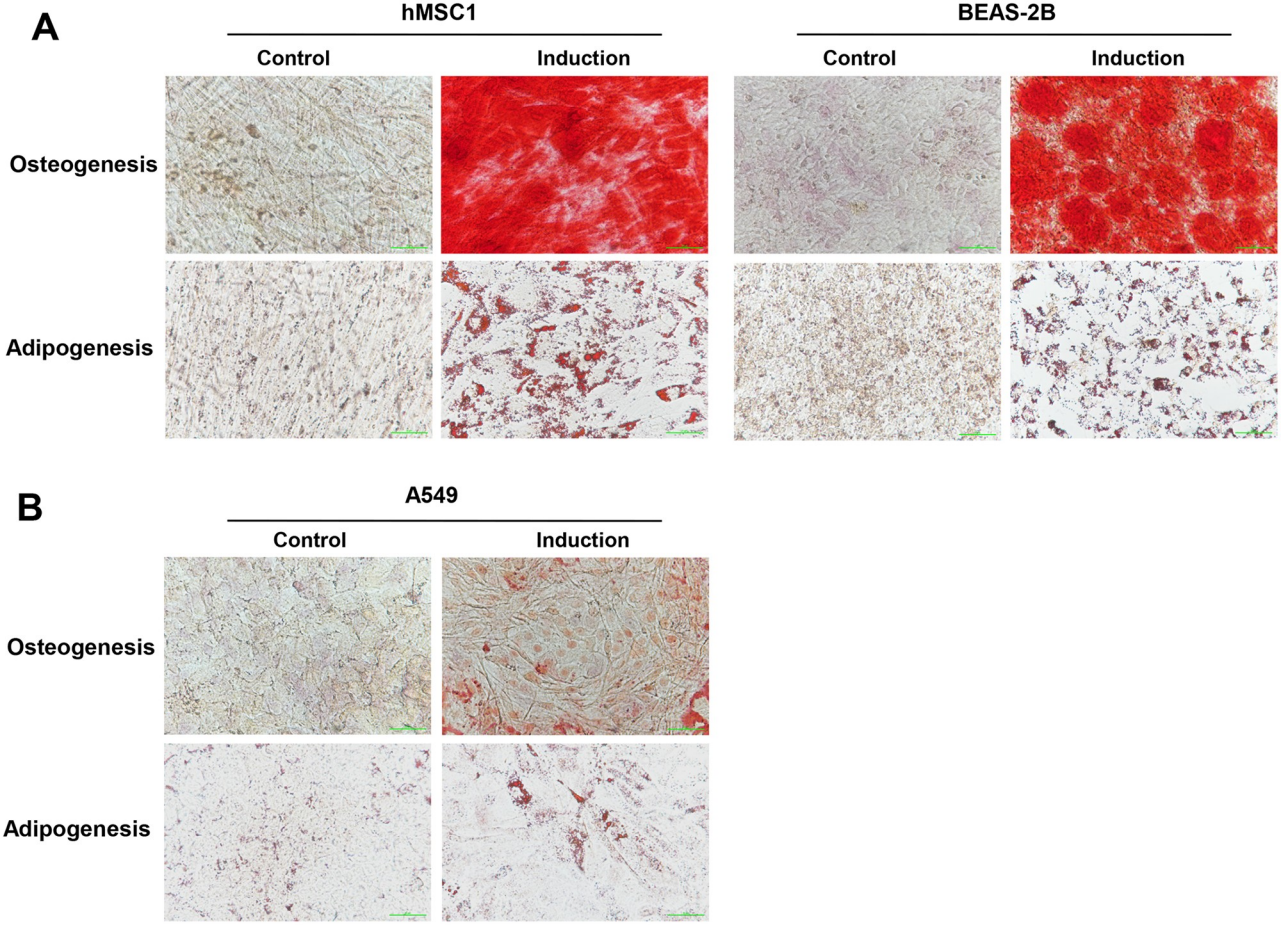
The BEAS-2B cells possess an equal differentiation potential for both osteogenesis and adipogenesis with hMSC1 cells. After culturing for 21 days under each differentiation induction condition, Oil Red O was used to stain lipid vacuoles in the differentiated adipocytes and Alizarin Red S was used to stain calcium deposits in the differentiated osteocytes. Both BEAS-2B and hMSC1 cells showed an almost equivalent positive staining for either osteogenesis or adipogenesis. (A). In comparison, the A549 cells exhibited a substantial staining for adipogenesis, but much weaker staining for osteogenesis (B). Scale bar = 50 μm.

Encouraged by the findings of expressing a full profile of surface markers of hMSCs, we then speculated that the BEAS-2B cells might also exhibit similarities with hMSCs on differentiation potentials and immunomodulatory activities. Next, we examined the *in vitro* ability of BEAS-2B cells to differentiate into osteocytes and adipocytes in comparison with hMSC1. The results showed that, similar to hMSC1, BEAS-2B exhibited a strong capability of differentiating into osteocytes and adipocytes after each differentiation induction (Fig 4A). In the meantime, we also examined the differentiation activities of both A549 and NCI-H1703 cells. It was found that, after differentiation induction, whereas the A549 cells exhibited a scarce differentiation potential for both adipocytes and osteocytes, the NCI-H1703 cells were resistant to both osteogenic and adipogenic differentiation as the cells died when exposing to the differentiation medium for longer than three days (Fig 4B). It was then suggested again that the BEAS-2B cells behaved more like hMSCs rather than lung epithelial cells.

### 5. The BEAS-2B cells shared the same immunomodulatory properties with hMSCs on inhibiting proliferation of both total T lymphocytes and Th1 subpopulation

**Fig 5:**
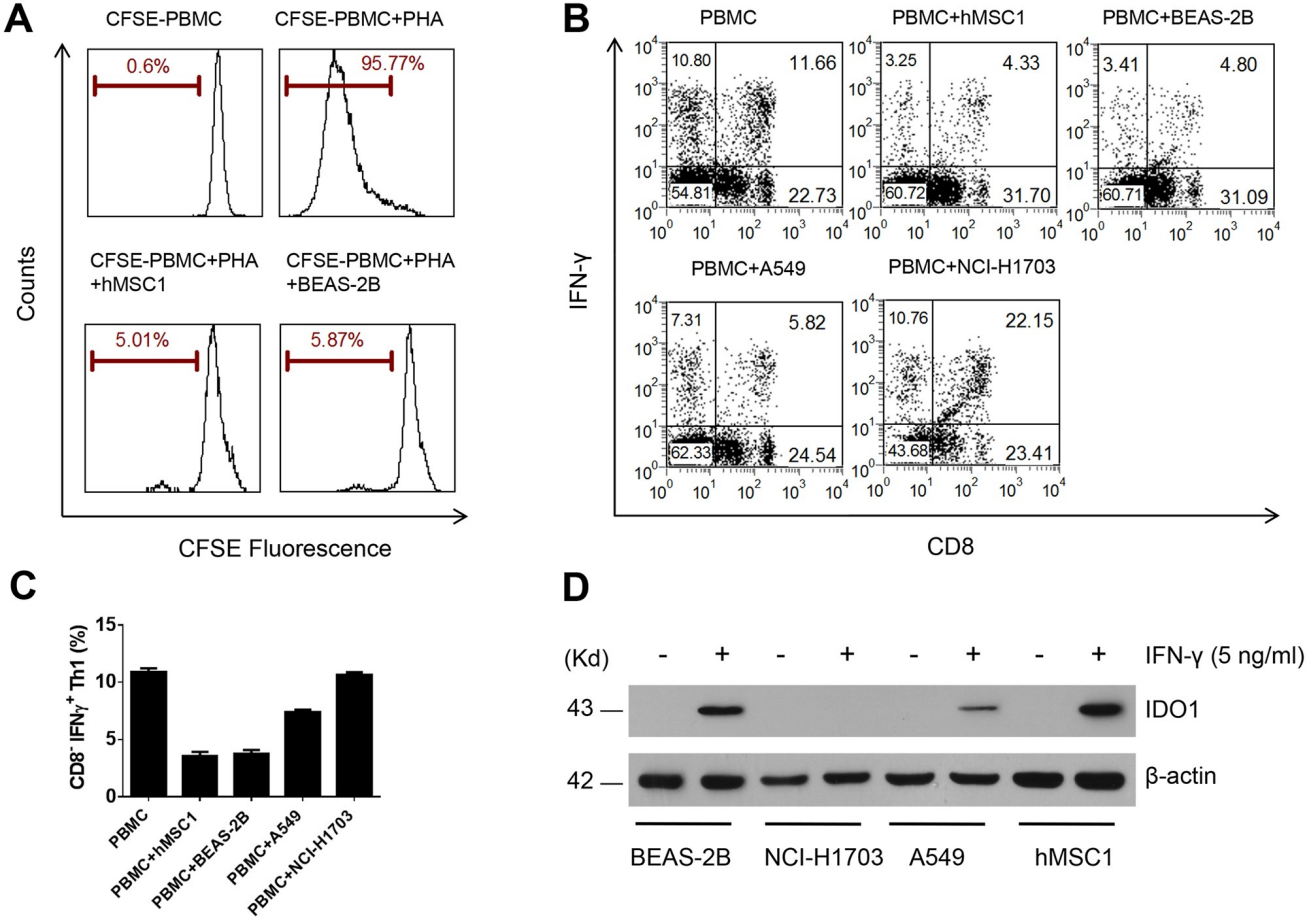
The BEAS-2B cells exhibit similar immunomodulatory activities with hMSC1 cells. In co-culturing with fresh PBMCs, both hMSC1 and BEAS-2B cells exhibit an equal inhibitory effect on proliferation of total lymphocytes labeled with CFSE (A), as well as Th1 lymphocytes (B). Whereas, the A549 cells exhibit a much lower inhibitory effect on Th1 lymphocytes than both BEAS-2B and hMSC1, and the NCI-H1703 cells show almost no such inhibitory effect, as seen in both flow cytometry dot-plot (B) and bar figure (C), which represent the results of three independent experiments. Western blotting shows that both hMSC1 and BEAS-2B express a high level of IDO1, whereas A549 express a lower level of IDO1 and NCI-H1703 does not express IDO1, following the treatment with IFN-γ. The expression of β-actin serves as the loading control in the test (D).

After testing the differentiation potential, we next examined the possible immunomodulatory activities of BEAS-2B cells in comparison with hMSC1. In the new experiments, the BEAS-2B cells were co-cultured with the PHA-activated PBMCs, then, either total T lymphocytes measured via a CFSE-based staining or the Th1 subgroup of CD4^+^ T lymphocytes represented by CD3^+^ CD8^-^ IFNγ^+^ lymphocytes were examined. Interestingly, we found that the BEAS-2B cells exerted a similarly significant inhibition on proliferation of both total T lymphocytes and Th1 lymphocytes (Fig 5A, 5B and 5C). Meanwhile, we also performed the co-culturing experiments to test the proliferation of Th1 lymphocytes for both A549 and NCI-H1703 cells. It was found that the A549 cells exhibited a much lower inhibitory effect on Th1 than both BEAS-2B and hMSC1, while the NCI-H1703 cells showed almost no such inhibitory effect (Fig 5B and 5C).

Since the indoleamine 2,3-dioxygenase 1 (IDO1) protein was believed to play a key role after activation by IFNγ or by other proinflammatory molecules in mediating immunomodulation by hMSCs, we next examined the IFNγ-induced IDO1 expression via Western blotting in BEAS-2B, A549 and NCI-H1703 cells in comparison with hMSC1. It was observed that both BEAS-2B and hMSC1 produced a tremendous amount of IDO1 after IFNγ induction, whereas the A549 cells produced a much lower amount, and the NCI-H1703 cells showed no detectable amount, of IDO1 expression (Fig 5D). Thus, the findings from the immunomodulation testing and IDO1 tests demonstrated that the BEAS-2B cells behaved again more like hMSCs than epithelial cells.

### 6. BEAS-2B did not exhibit promoting effect on M2 macrophage polarization

**Fig 6:**
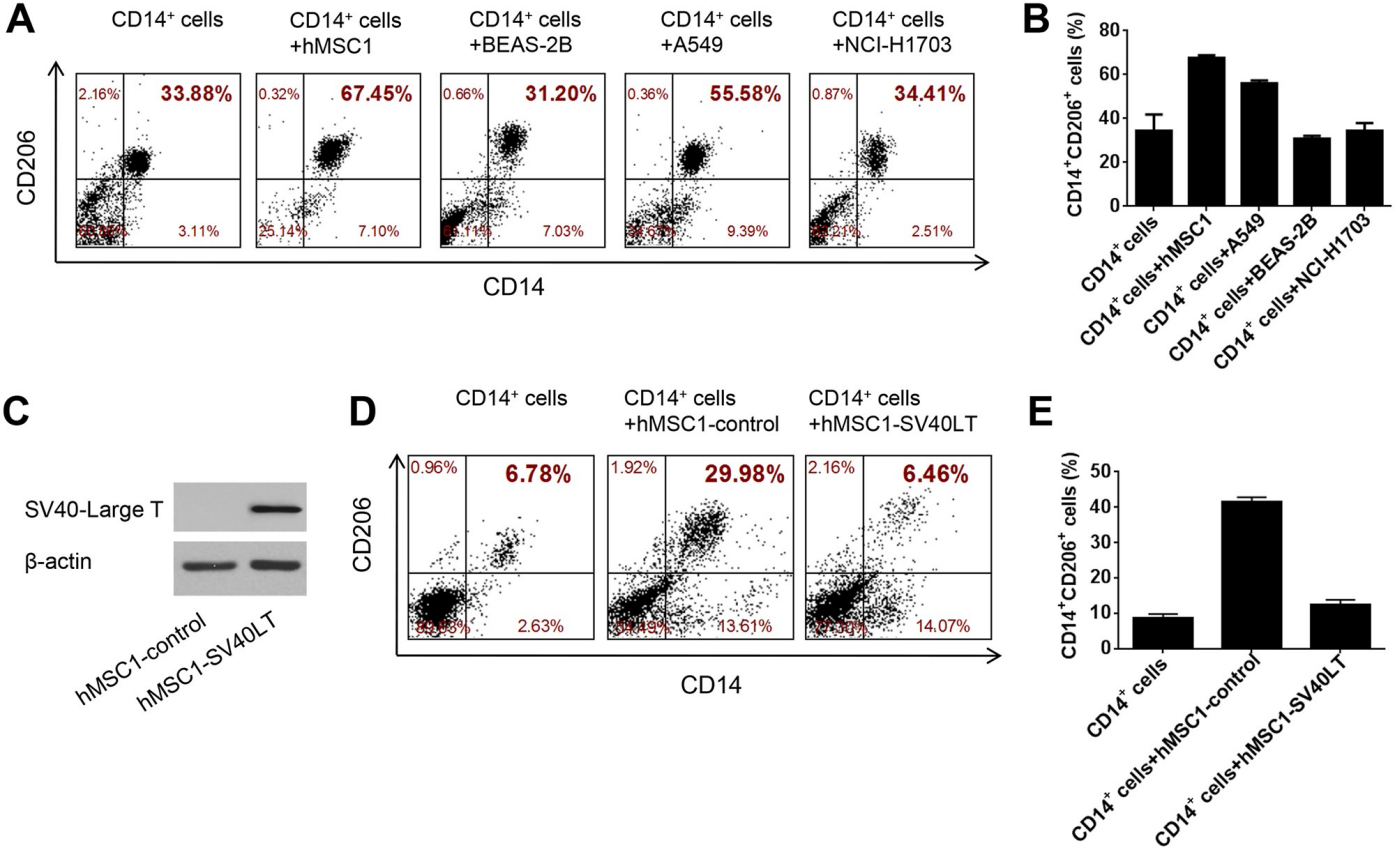
The BEAS-2B cells do not promote M2-macrophage polarization. In co-culturing with CD14^+^ monocytes purified from fresh PBMCs, hMSC1 dramatically elevate the proportion of CD14^+^/CD206^+^ M2 macrophages; whereas BEAS-2B and NCI-H1703 cells show no such effect, and A549 cells exhibited a moderate elevation of M2 macrophages, as seen both in flow cytometry dot-plot (A) and bar figure (B). The hMSC1-SV40LT cells stably transfected with SV40-LT express SV40-LT protein as tested by Western Blotting in comparison with hMSC1-control cells (C). In co-culturing with CD14^+^ monocytes purified from fresh PBMCs, whereas the hMSC1-control cells induce a dramatic increase in the proportion of CD14^+^/CD206^+^ M2 macrophages, the hMSC1-SV40LT cells do not show such effect, as seen both in flow cytometry dot-plot (D) and bar figure (E), both of which represent the results of three independent experiments.

After demonstrating the shared activities by BEAS-2B and hMSC1 on T lymphocytes, we next conducted a separate co-culturing experiment to reveal the possible activity of BEAS-2B in modulating macrophage polarization/differentiation. We first isolated human CD14^+^ monocytes from fresh PBMCs and then co-cultured the monocytes with BEAS-2B cells or hMSC1. At the end of the co-culturing, we examined the changes in expression of CD206, a common surface marker of all types of M2 macrophages, in CD14^+^ cells [29]. It was shown that hMSC1 induced a dramatic increase in the proportion of CD14^+^/CD206^+^ M2 macrophages; whereas BEAS-2B cells did not show such an effect (Fig 6A and 6B). In the meantime, we also conducted the same assay for both A549 cells and NCI-H1703 cells. The new findings showed that, like BEAS-2B cells, NCI-H1703 did not promote M2 macrophage polarization, while A549 showed a moderate capability of inducing M2 polarization (Fig 6A and 6B).

Given the original report as well as our validated finding that BEAS-2B was positive for SV40 LT antigen (Fig 1B), we then attempted to interpret that the failed inhibition of BEAS-2B on M2 macrophage polarization could be attributable to the expression of SV40 Large T antigen. To test this hypothesis, we performed a lentivirus-mediated transfection on hMSC1 to derive new hMSC1-SV40LT cells stably expressing SV40 LT antigen (Fig 6C), and then examined the effect of hMSC1-SV40LT cells on M2 macrophage polarization in comparison with hMSC1-Control transfected with empty vector. As a result, the hMSC1-SV40LT cells became almost incapable of promoting M2 polarization (Fig 6D and 6E), thus supporting the hypothesis that the incapability of BEAS-2B on M2 macrophage polarization was a consequence of the expression of SV40 LT antigen.

### 7. The mesenchymal properties of BEAS-2B cells was not likely a consequence of EMT

**Fig 7:**
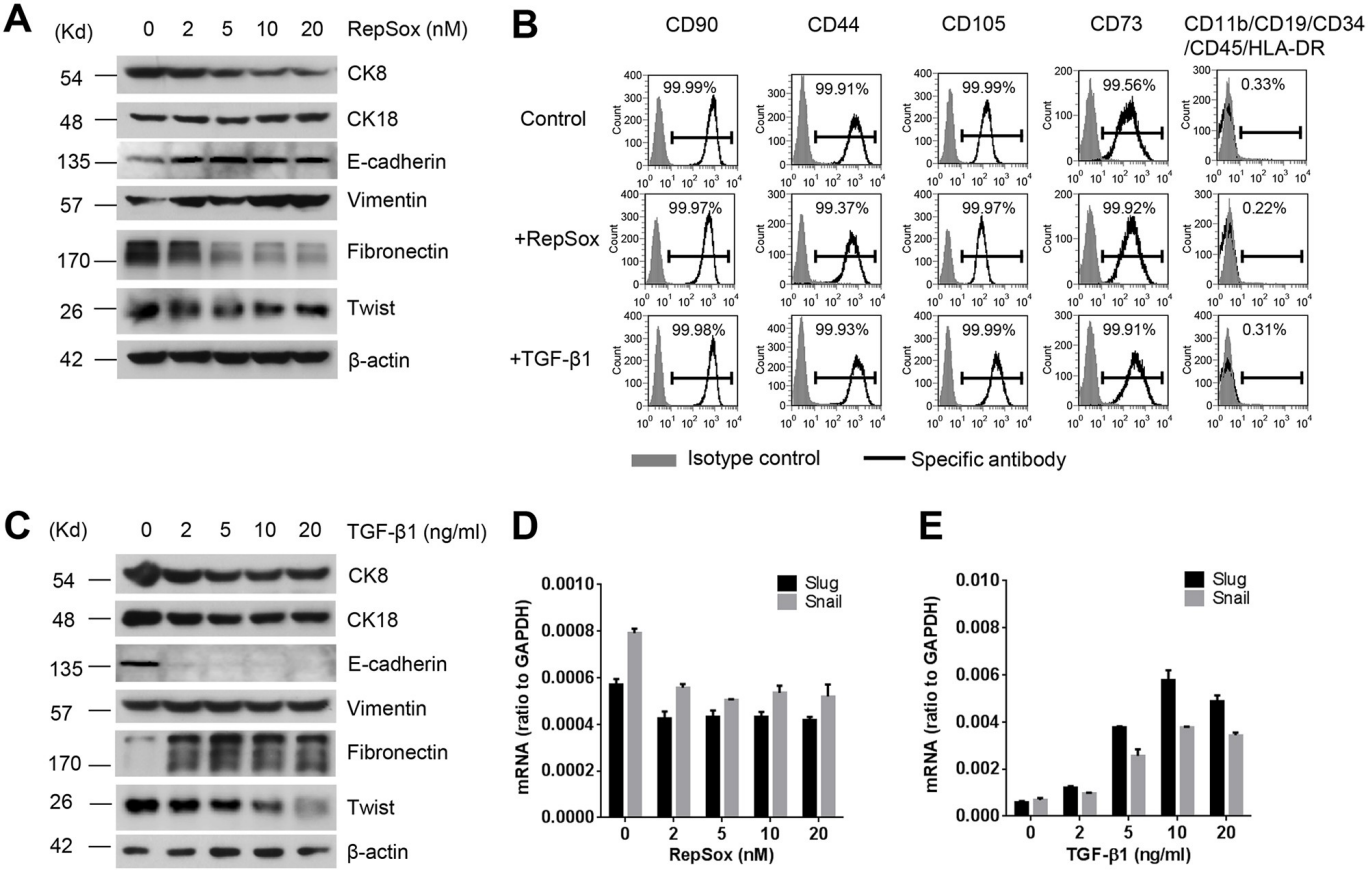
Epithelial mesenchymal transition (EMT) is not a contributing factor to the mesenchymal properties of BEAS-2B cells. Western blotting shows that the treatment with RepSox for 5 days induced a reduction in expression of CK8 and Fibronectin, and an elevation in vimentin and E-cadherin, but did not induce significant changes in expression of CK18 and Twist. The expression of β-actin served as the loading control in the test (A). The treatment with RepSox or TGF-β1 for 5 days did not affect the expression of MSC surface markers in BEAS-2B cells, as determined by flow cytometry (B). Western blotting reveals that the treatment with TGF-β1 for 5 days did not affect the expression of CK8, CK18 and vimentin, but increased fibronectin and decreased E-cadherin and Twist. The expression of β-actin served as the loading control in the test (C). The quantitative RT-PCR showed that the RepSox treatment did not elicit dose-dependent changes in the transcription of Slug and Snail (D), however, the TGF-β1 treatment elevated the transcription of Slug and Snail in a dose dependent manner (E).

Given the similarities shared by BEAS-2B and hMSC1 in surface marker profile, differentiation potential, and immunomodulatory activities, it was thus concluded that the BEAS-2B cells exhibited an almost identical profile of hMSCs. However, it was unclear whether the exhibited properties were originally possessed before cell line derivation or acquired during long *in vitro* cell culturing/passaging. Considering that BEAS-2B has served as an extremely popular cell line in various *in vitro* studies, it was not impossible that the mesenchymal properties of the cells could be acquired via the mechanism of EMT during long *in vitro* culturing even its origin was of epithelial nature. If the EMT happened to be the case, it could be most likely induced by TGF-β1, which exists in fetal bovine serum in most *in vitro* cell culture system, as the TGF-β1 signaling represents the most well-known inducer of EMT [30]. Next, we attempted to determine whether the EMT could be the contributing mechanism to the acquisition of mesenchymal properties of BEAS-2B by consecutively treating the cells with either TGF-β1 or RepSox, a selective TGF-β1 signaling inhibitor, before analyzing the cells for the mesenchymal properties. Unexpectedly, after consecutive treatment for 5 days with 2–20 nM RepSox, a dosage range reported previously of being able to inhibit TGF-β1 signaling activity and subsequent inhibition of EMT [31], the expression of CK8 and fibronectin, a epithelial marker and a common matrix protein of fibroblasts, respectively, was significantly reduced, whereas the expression of mesenchymal marker vimentin was elevated, and all these changes were clearly in a dose-dependent manner (Fig 7A). The RepSox treatment also induced an elevation in E-Cadherin, but this effect was not dose-dependent. In the meantime, the RepSox treatment did not cause significant changes in expression of epithelial marker CK18 as well as Twist, Slug, and Snail, which are the three key transcriptional factors involved in TGF-β signaling and EMT, as detected by Western blotting (Fig 7A) or quantitative RT-PCR (Fig 7D). Most importantly, RepSox did not induce any change on CD73, CD44, CD90, CD105, the key hMSCs surface markers (Fig 7B).

In a parallel experiment, the treatment with 2-20ng/ml TGF-β1 did not induce any change in the expression of CK8, CK18, and vimentin, but showed a significant increase in fibronectin and a reduction in E-cadherin (Fig 7C). Although the expression of both Slug and Snail, as detected by quantitative RT-PCR, was elevated, the expression of Twist, as examined by Western blotting, was dramatically reduced in a dose-dependent manner after the TGF-β1 treatment (Fig 7C and 7E). Similar to the RepSox treatment, no changes were observed in CD73, CD44, CD90, CD105 following the treatment with TGF-β1 (Fig 7B). Thus, all the observations did not support the speculation that the mesenchymal properties of BEAS-2B were acquired through the mechanism of EMT.

## Discussion

Epithelial cells and mesenchymal cells are two cell types in terms of cell biology properties developed along embryonic differentiation. Both cell types co-exist in almost all types of tissues including epithelial tissues. There is always technical difficulty to separate epithelial cells from mesenchymal cells during isolation and establishment of primary epithelial cells. Therefore, without extensive characterization, a newly established cell line could be easily wrongly recognized for its cell type identity. Based on the present observations, the BEAS-2B cell line may represent such an example of wrong recognition.

The BEAS-2B cell line was originally established as a human non-tumorigenic lung epithelial cell line derived from a human lung tissue and has been extensively used as an *in vitro* non-tumorigenic lung epithelial model in a large variety of studies in association with lung carcinogenesis for over 30 years. However, very few studies have challenged its non-epithelial properties since the cell line was originally accepted as an epithelial cell line following the initial evidence to support the epithelial origin. In the present study, we have extended characterization for this cell line to pursue its mesenchymal properties. After a comprehensive comparison with hMSCs in both phenotypes and biological functions, we demonstrated for the first time that BEAS-2B cells exhibited an almost identical profile of hMSCs. It was further believed that the mesenchymal features of BEAS-2B are of intrinsic nature rather than acquired through the process like EMT during long *in vitro* exposure to TGFβ1-containing cell culture system.

At the beginning, our new investigations for mesenchymal properties of BEAS-2B were encouraged by an unintentional finding that BEAS-2B expressed a group of definitive surface markers of hMSCs, which was proposed by ISCT in 2006 as part of a minimal criteria for defining hMSCs of different tissue origins [16]. The BEAS-2B cell line used in our experiments has been validated as a non-tumorigenic and SV40-LT expressing cell line with an identical fingerprint to the same cell line deposited in the ATCC.

From the characterization of marker proteins of both mesenchymal cells and epithelial cells, BEAS-2B was found to be identical to hMSC1 in the profile of all surface marker proteins used to define hMSCs. Meanwhile, the surface marker profiles of NCI-H1703 and A549 cells were largely different from both BEAS-2B and hMSC1, particularly different in CD105 (Fig 3). In addition to the surface markers, the BEAS-2B cells also expressed CK8 and CK18, which were assumed to be epithelial marker proteins in original report [9], but did not express α-SMA, fibronectin and collagen I, which are the proteins abundant in mesenchymal cells [32]. However, it was found in our studies that CK8 and CK18 were not unique to epithelial cells as hMSC1 also expressed these two proteins (Fig 1B) and several other hMSCs cell lines from different donors in our studies expressed CK8 and CK18 as well (S1 Fig). A similar situation was also seen in the expression of EpCAM, which was found positive in BEAS-2B and A549 cells, but negative in NCI-H1703 cells (Fig 1D). In addition, the undetectable expression of α-SMA, fibronectin and collagen I in BEAS-2B was not likely to exclude the possibility of its mesenchymal features because BEAS-2B was established from the transformation by SV40-LT, which was found from our limited observations to be able to suppress the expression of α-SMA, fibronectin and collagen I proteins in hMSCs cells including hMSC1 (S2 Fig). Nevertheless, the identical profile to the MSCs surface markers strongly supports the possession of mesenchymal features of BEAS-2B.

From the characterization on multi-lineage differentiation potential, BEAS-2B and hMSC1 were found to share an almost identical potential in both osteogenic and adipogenic differentiation, whereas A549 exhibited a substantial potential for adipogenesis, but much lower potential for osteogenesis, whereas the NCI-H1703 cells possessed almost no potential for both of them (Fig 4). The difference existing between A549 and NCI-H1703 may be interpreted by the difference in malignancy; low malignant epithelial cells, like NCI-H1703, possess no or very low differentiation potential, and high malignant epithelial cells, such as A549, behave more like cancer stem cells thus exhibiting certain level of differentiation potential. Since A549 was not a *de novo* MSCs line, its differentiation potential is believed to be acquired during its malignant progression. But even though, the differentiation potential of A549 should be much lower than that of hMSC1. Nevertheless, the findings on the equivalence in differentiation potential between BEAS-2B and hMSC1 add a separate piece of evidence to support the possession of mesenchymal stem cell features by BEAS-2B.

From the characterization on immunomodulatory activities, the BEAS-2B cells also exhibited an almost identical immunomodulation profile with hMSC1. In comparison, the immunomodulation profile for A549 and NCI-H1703 was much different from both BEAS-2B and hMSC1. Whereas the A549 cells exhibited a much lower capability of inhibiting the activated lymphocytes and a much lower production of the IFNγ-induced IDO1 than both BEAS-2B and hMSC1, the NCI-H1703 cells showed an even lower ability than A549 to suppress the activated lymphocyte proliferation and no expression of the IFNγ-induced IDO1. Following the same logic, the difference in immunomodulation profile between A549 and NCI-H1703 could also be interpreted by the difference in malignancy between the two cancer lines, in which A549 represented a more malignant cells having acquired a certain level of immunomodulation, which further contributed to the increased malignancy of the cells, while the NCI-H1703 cells had not progressed sufficiently to acquire the capability of immunomodulation.

In summary, with the new evidence showing almost identical profiles in surface markers, differentiation potential and immunomodulation between hMSC1 and BEAS-2B, it is thus believed that the BEAS-2B cells should be accepted as a *bona fide* mesenchymal stem cells.

While considering the possibility of intrinsically possessed mesenchymal features by BEAS-2B, it needs to exclude the possibility that the mesenchymal features of BEAS-2B could be acquired during long *in vitro* exposure to the factors that promote EMT as the EMT has been well accepted as a common mechanism for epithelial cells to acquire mesenchymal features [33, 34]. TGFβ1 represents a well-characterized EMT inducer and exists in FBS in large amount. As the BEAS-2B has served as a very common *in vitro* cell model used frequently in various *in vitro* studies and the medium for growing BEAS-2B has long been changed from serum-free medium to FBS-containing medium, it is thus necessary to exclude the possibility that the mesenchymal features of BEAS-2B could be acquired after long *in vitro* exposure to TGFβ1-containing FBS via the process of EMT [30]. Indeed, the new results from the experiments using TGFβ1 or RepSox, did not fully support the possibility of EMT, as both the supporting and non-supporting evidence to the mechanism of EMT were seen. Most importantly, the treatment with either RepSox or TGF-β1 did not induce any changes in expression of CD73, CD44, CD90, and CD105, the definitive hMSCs surface markers. These findings thus excluded the EMT mechanism, meanwhile, further supported that BEAS-2B was of intrinsic rather than acquired mesenchymal features. The possible wrong recognition of BEAS-2B as an epithelial cell line could be very likely an outcome of incomplete characterization at the time of cell line establishment and no further characterization thereafter.

If the BEAS-2B is eventually validated as an essential hMSC cell line, it would be necessary to review the designs and results derived from a large amount of studies reported previously using BEAS-2B as an *in vitro* cell model, and all the findings based on the assumption of epithelial essence of BEAS-2B should be re-directed to the findings associated with hMSCs features. From the points of MSCs, the newly revealed mesenchymal features of BEAS-2B may be of great importance in the field of hMSCs studies.

MSCs has emerged as the most frequent type of stem cells used in both preclinical and clinical stages of stem cell therapies [35, 36]. However, the quality evaluation of hMSCs still remains to be a big challenge mainly because of the lack of stable hMSCs reference cell line [37]. Various quality factors within the quality evaluation testing, such as the quality of PBMCs used for evaluating immunomodulatory activities of hMSCs, the quality of testing reagents and testing medium, may affect quality evaluation of hMSCs products [37]. Therefore, to eliminate the interferences from various factors, it is extremely important to establish a reference cell line. BEAS-2B may represent such a reference cell line.

In conclusion, the present study demonstrated for the first time that BEAS-2B cells line may represent a *bona fide* hMSCs cell line rather than epithelial cell line. Further validation tests are warranted for clarifying its intrinsic mesenchymal identity as well as for reinterpreting previous findings achieved from using BEAS-2B as an *in vitro* epithelial model. Meanwhile, the mesenchymal properties may give BEAS-2B a unique advantage over primary hMSCs for being a valuable hMSCs cell line in the field of quality control studies of hMSCs.

## Supporting information

S1 Fig hMSCs cell lines from different donors consititutively express CK8 and CK18. Cell lysates of hMSCs cell lines from 7 different donors, including hMSC1, were prepared and detected by Western Blot for expression of epithelial markers CK8 and CK18. Expression of β-actin served as the loading control in the test.
(TIF)

S2 Fig SV40-LT transformation could suppress the expression of mesenchymal markers in hMSCs. hMSC1 was stably transfected with control vector (hMSC1-control), hTERT (hMSC1-hTERT), or SV40-LT (hMSC1-SV40LT) by lentivirus. Cell lysates from hMSC1, hMSC1-control, hMSC1-hTERT, and hMSC1-SV40LT were collected and detected by Western Blot for expression of α-SMA, collagen I, fibronectin, and vimentin. Expression of β-actin served as the loading control in the test. Unlike hTERT immortalization, SV40-LT transformation could suppress the expression of mesenchymal markers in hMSC1.
(TIF)

S1 Raw Images (PDF)
