# Supplementary figures and images for: Human lung epithelial BEAS-2B cells exhibit characteristics of mesenchymal stem cells
Source: PLoS One. 2020 Jan 3;15(1):e0227174. doi: 10.1371/journal.pone.0227174 (PMC6941928; doi:10.1371/journal.pone.0227174)

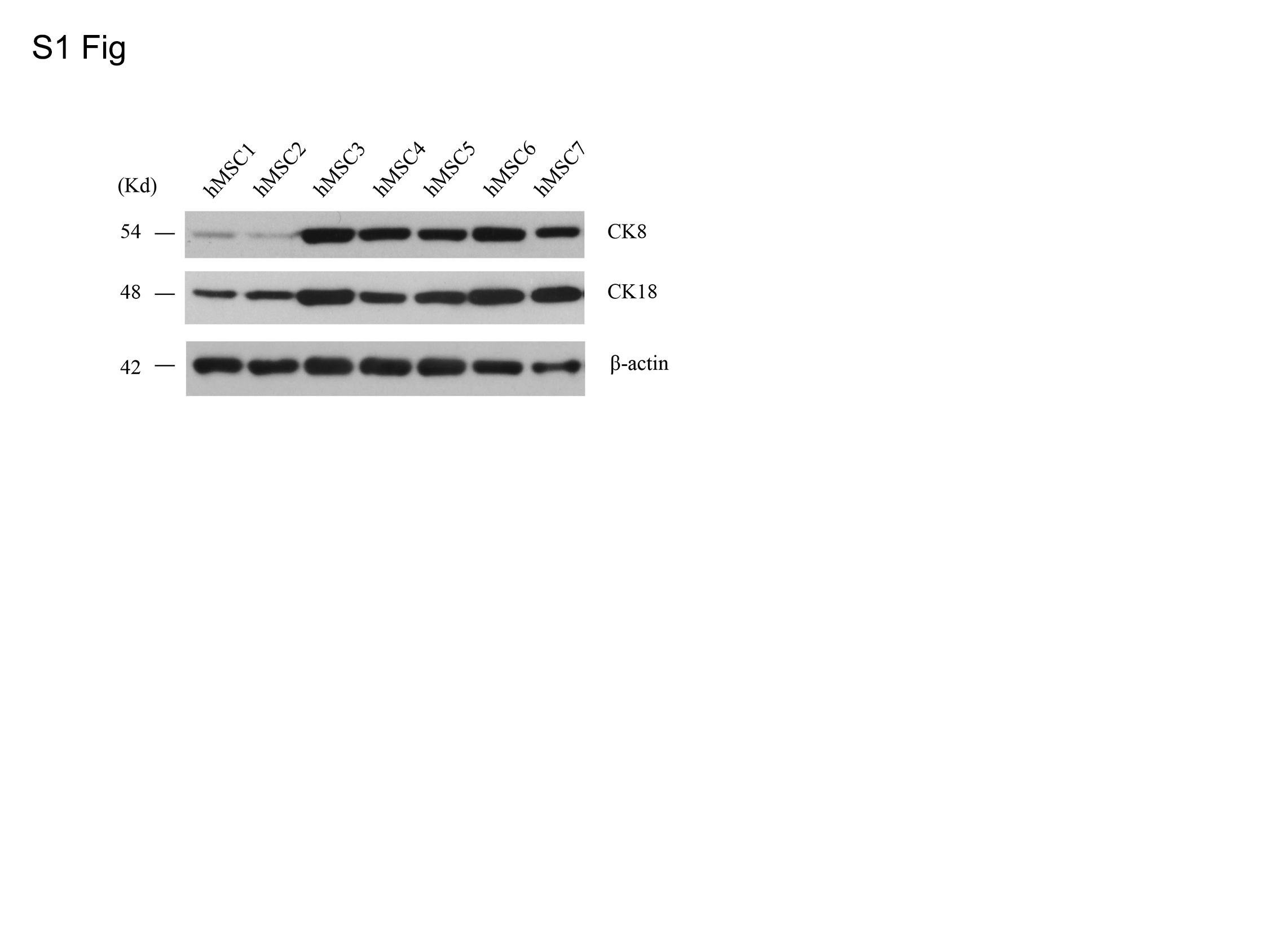

Supplement: S1 Fig — Cell lysates of hMSCs cell lines from 7 different donors, including hMSC1, were prepared and detected by Western Blot for expression of epithelial markers CK8 and CK18. Expression of β-actin served as the loading control in the test. (TIF) [file pone.0227174.s001.tif]

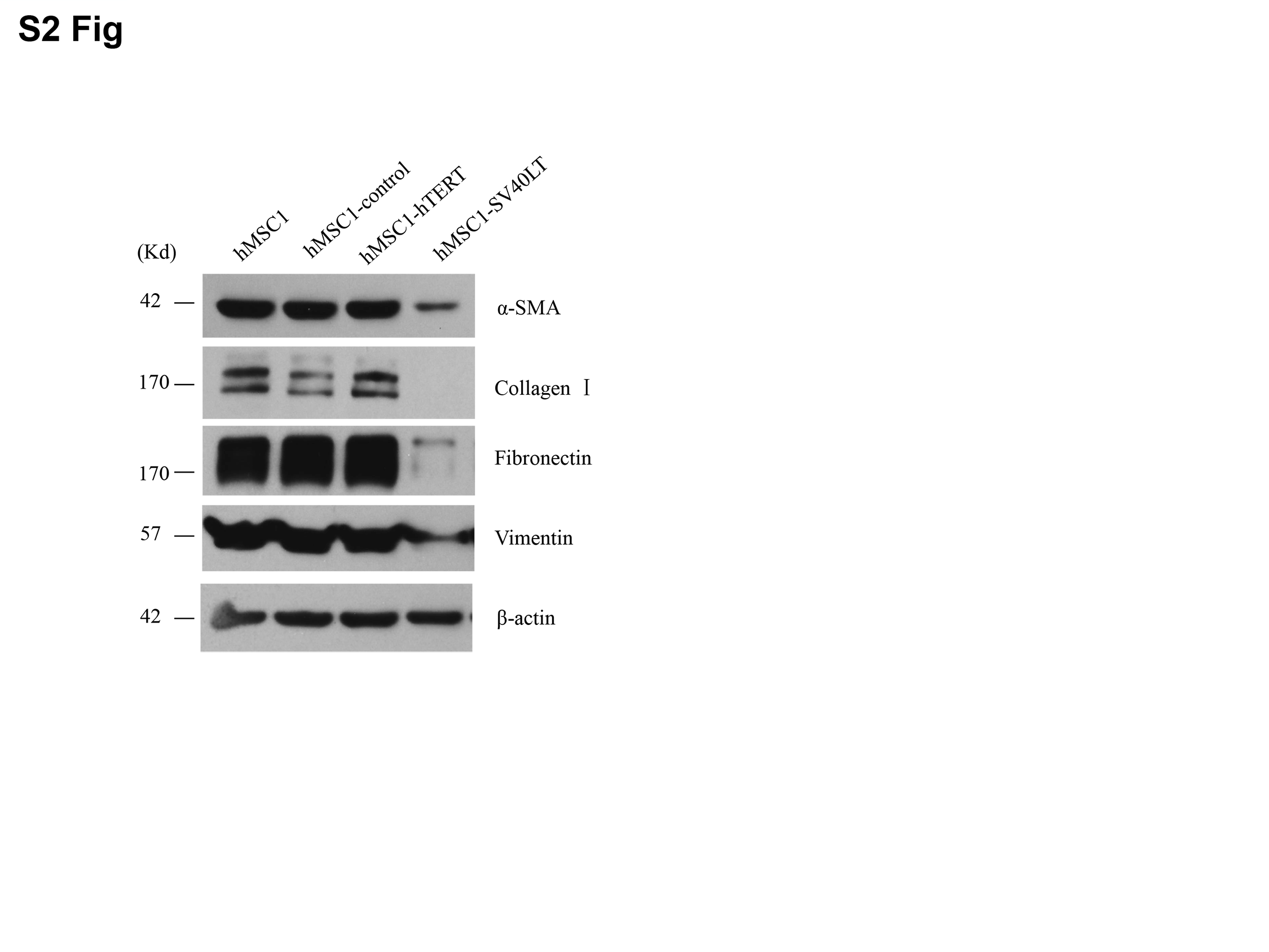

Supplement: S2 Fig — hMSC1 was stably transfected with control vector (hMSC1-control), hTERT (hMSC1-hTERT), or SV40-LT (hMSC1-SV40LT) by lentivirus. Cell lysates from hMSC1, hMSC1-control, hMSC1-hTERT, and hMSC1-SV40LT were collected and detected by Western Blot for expression of α-SMA, collagen I, fibronectin, and vimentin. Expression of β-actin served as the loading control in the test. Unlike hTERT immortalization, SV40-LT transformation could suppress the expression of mesenchymal markers in hMSC1. (TIF) [file pone.0227174.s002.tif]
