# Supplementary material for: Human lung epithelial BEAS-2B cells exhibit characteristics of mesenchymal stem cells
Source: PLoS One. 2020 Jan 3;15(1):e0227174. doi: 10.1371/journal.pone.0227174 (PMC6941928; doi:10.1371/journal.pone.0227174)
Supplement: S1 Raw Images — (PDF) [file pone.0227174.s003.pdf]

In the following raw images, all the relevant data contained in the figures and supplemental figures, were marked with red rectangles.

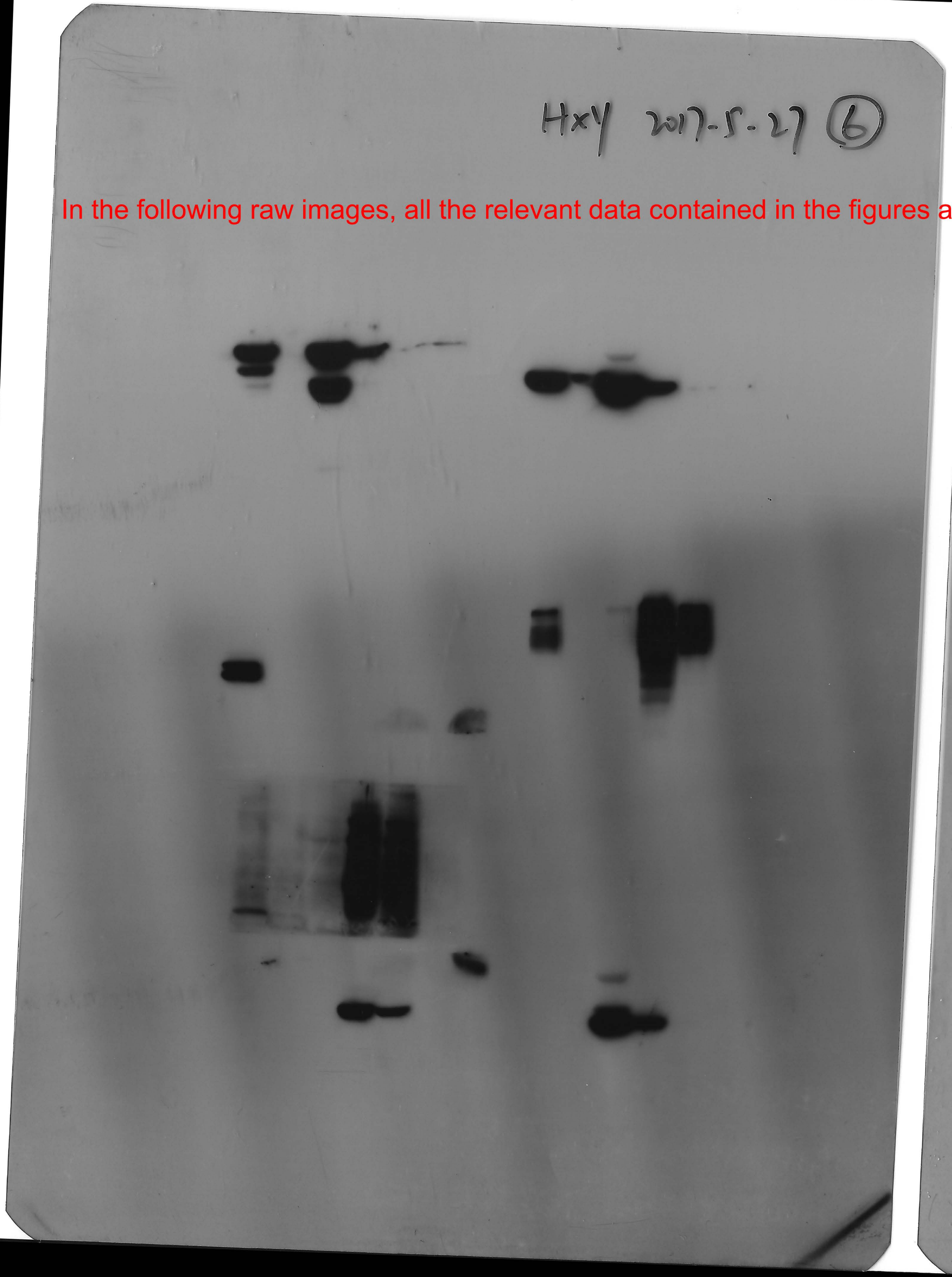

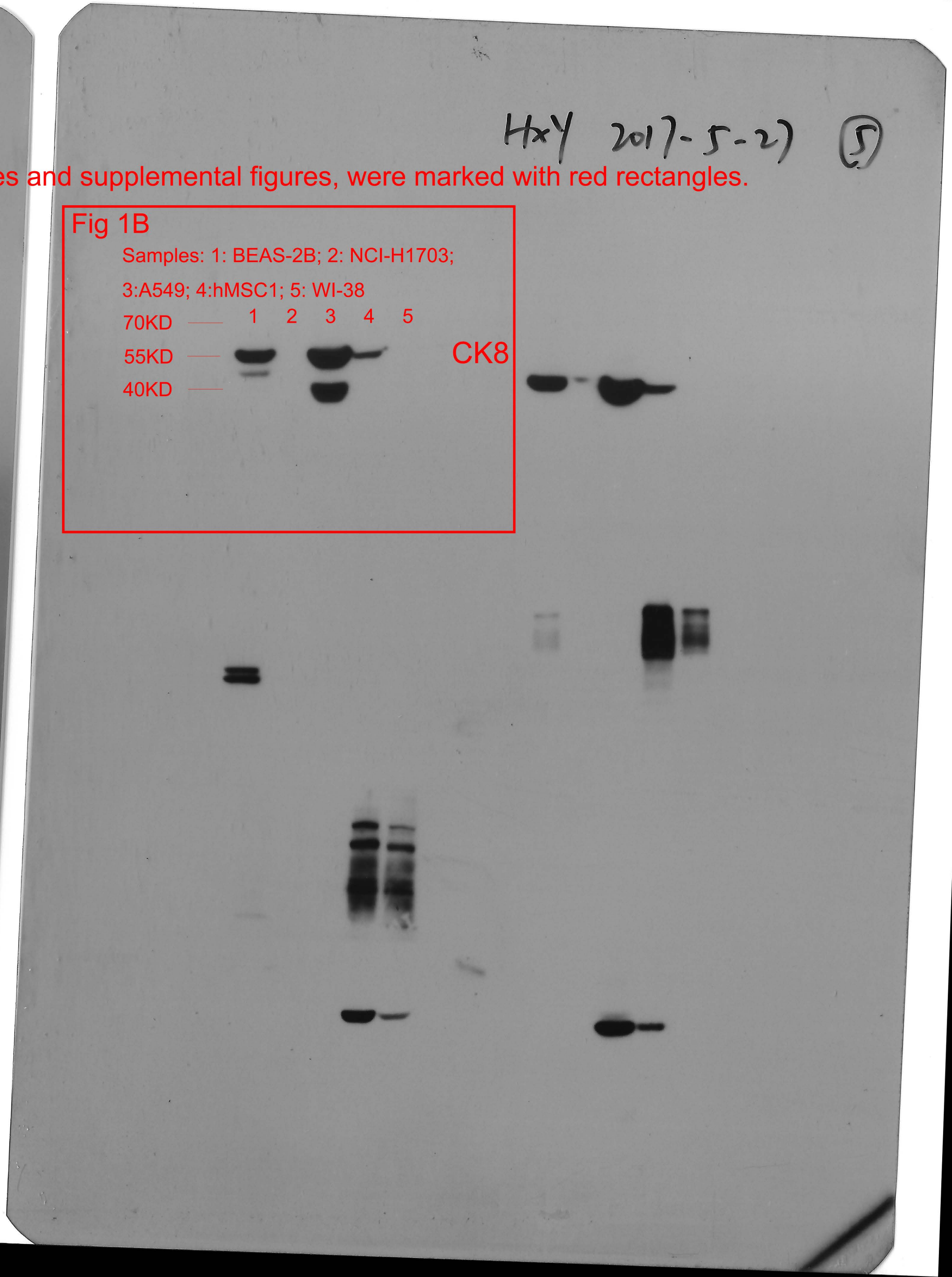

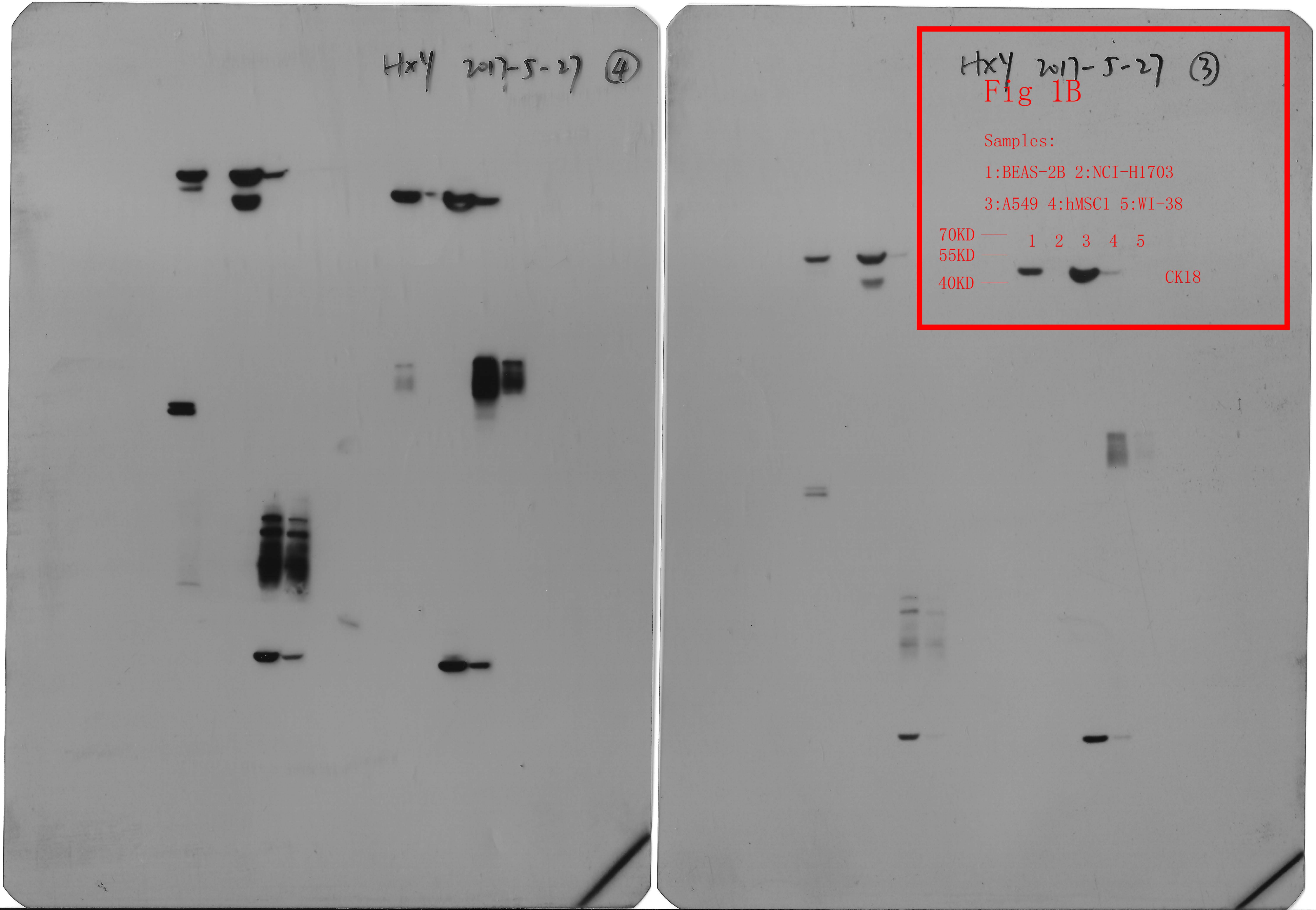
Hxy 2017-5-27 ④
Hxy 2017-5-27 ③
Fig 1B
Samples:
1:BEAS-2B 2:NCI-H1703
3:A549 4:hMSC1 5:WI-38
70KD
55KD
40KD
1 2 3 4 5
CK18

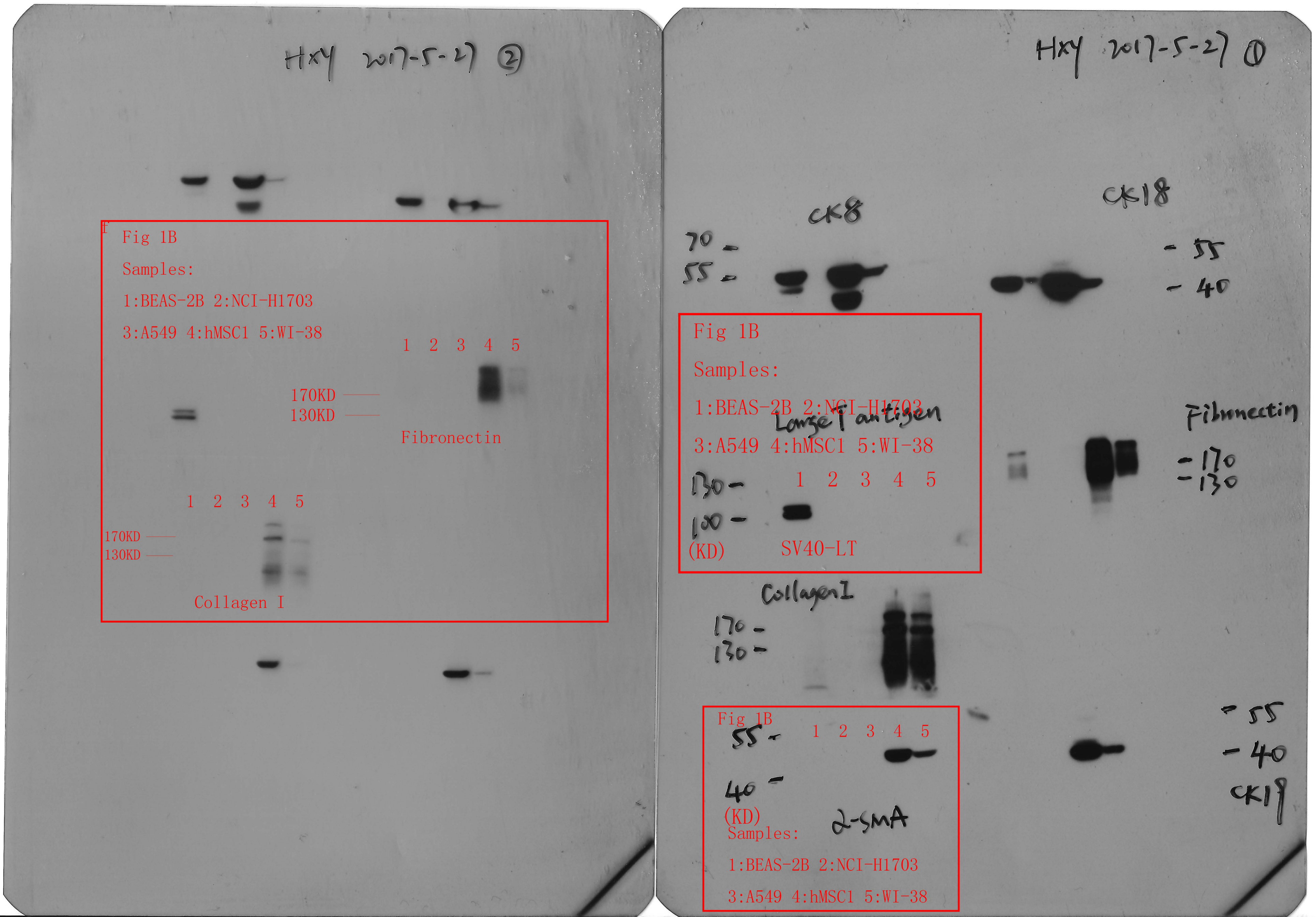
Hxy 2017-5-27 ②
Fig 1B
Samples:
1:BEAS-2B 2:NCI-H1703
3:A549 4:hMSC1 5:WI-38
1 2 3 4 5
170KD
130KD
Fibronectin
1 2 3 4 5
170KD
130KD
Collagen I
Hxy 2017-5-27 ①
CK8
CK18
70
55
55
40
Fig 1B
Samples:
1:BEAS-2B 2:NCI-H1703
Large T antigen
3:A549 4:hMSC1 5:WI-38
130
1 2 3 4 5
100
(KD)
SV40-LT
Fibronectin
170
130
Collagen I
170
130
Fig 1B
55
1 2 3 4 5
55
40
40
CK19
(KD)
α-SMA
Samples:
1:BEAS-2B 2:NCI-H1703
3:A549 4:hMSC1 5:WI-38

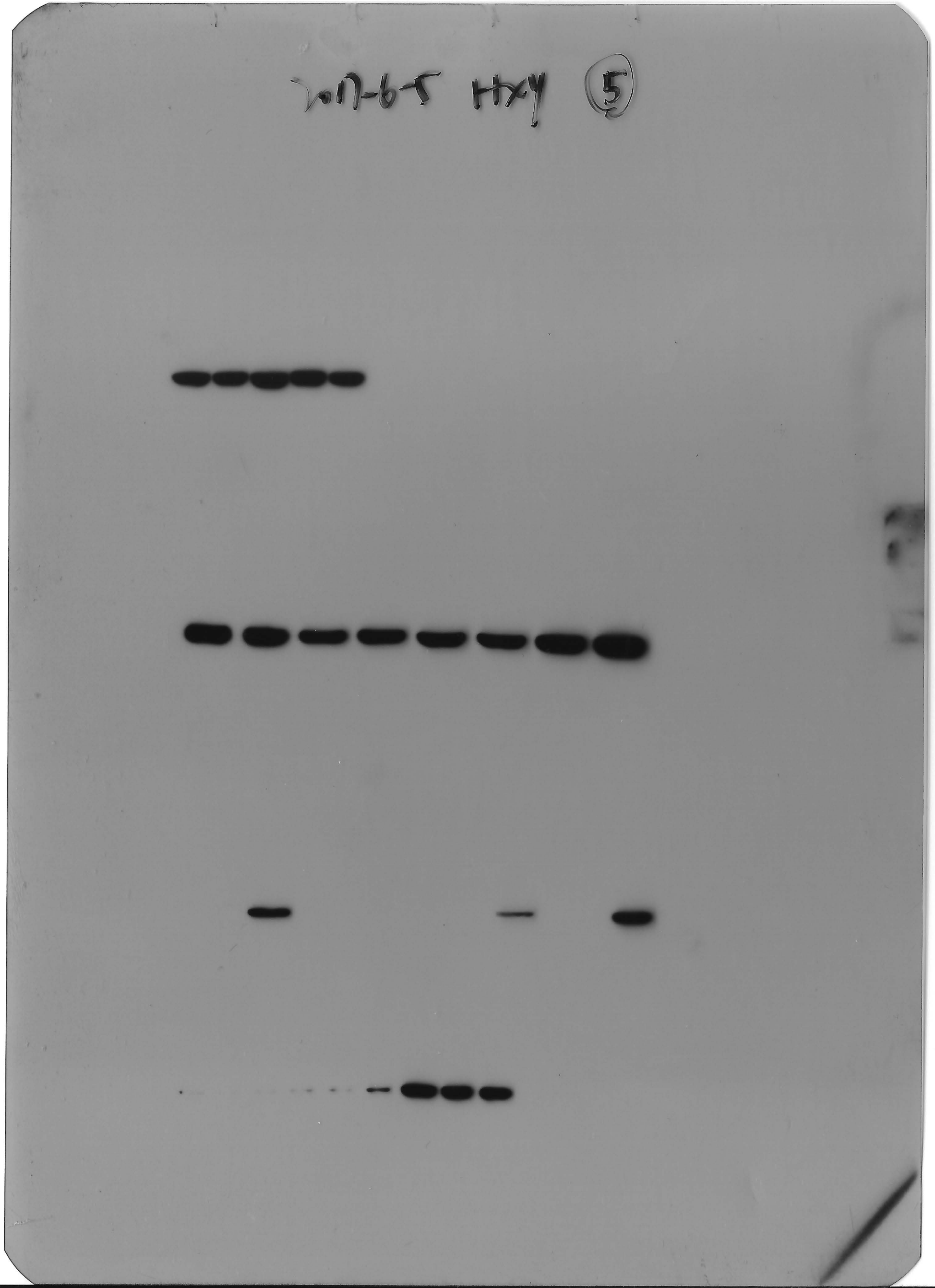
2017-6-5 Hxy 5

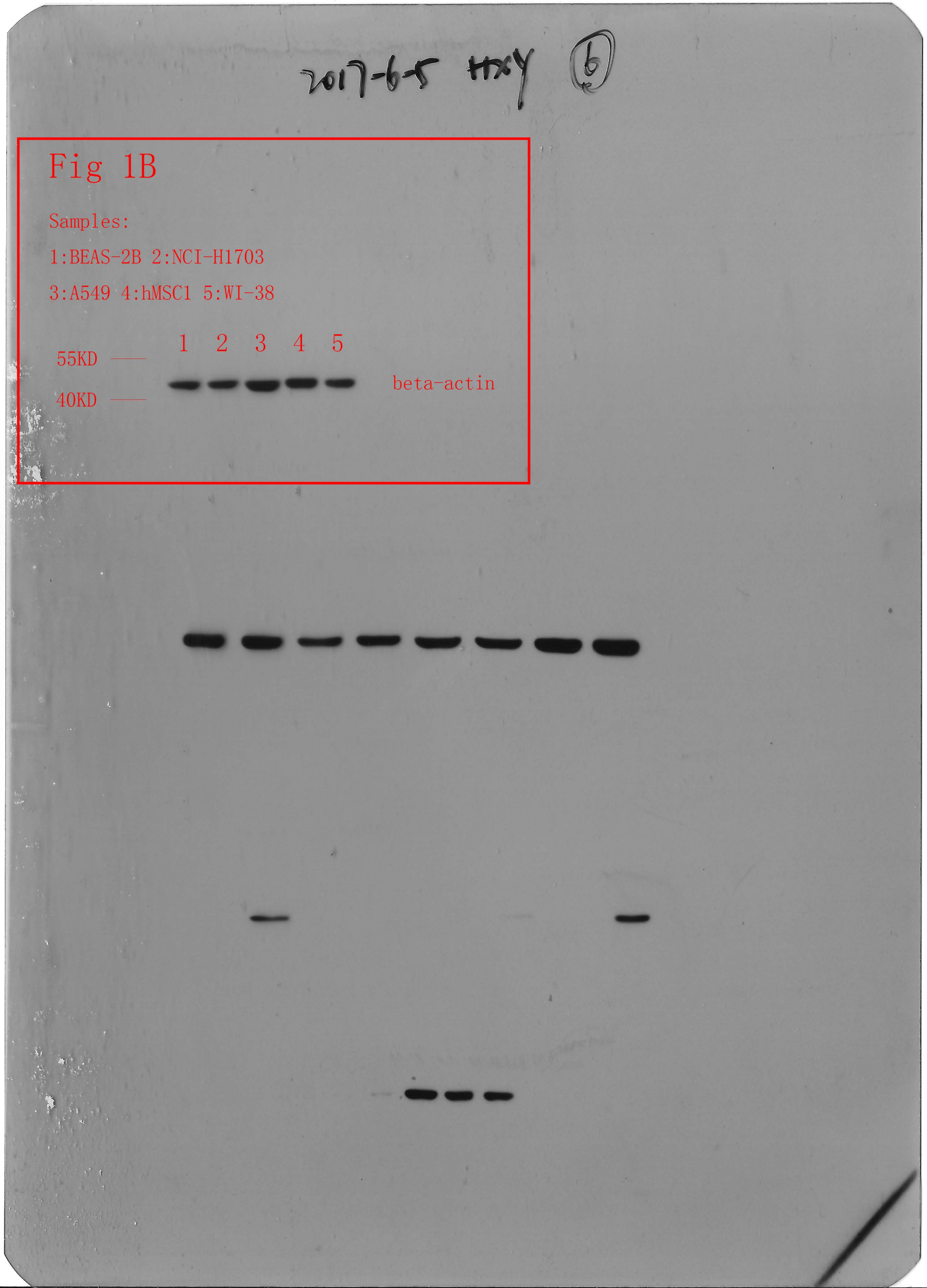
2017-6-5 Hxy 6
Fig 1B
Samples:
1:BEAS-2B 2:NCI-H1703
3:A549 4:hMSC1 5:WI-38
1 2 3 4 5
55KD
40KD
beta-actin

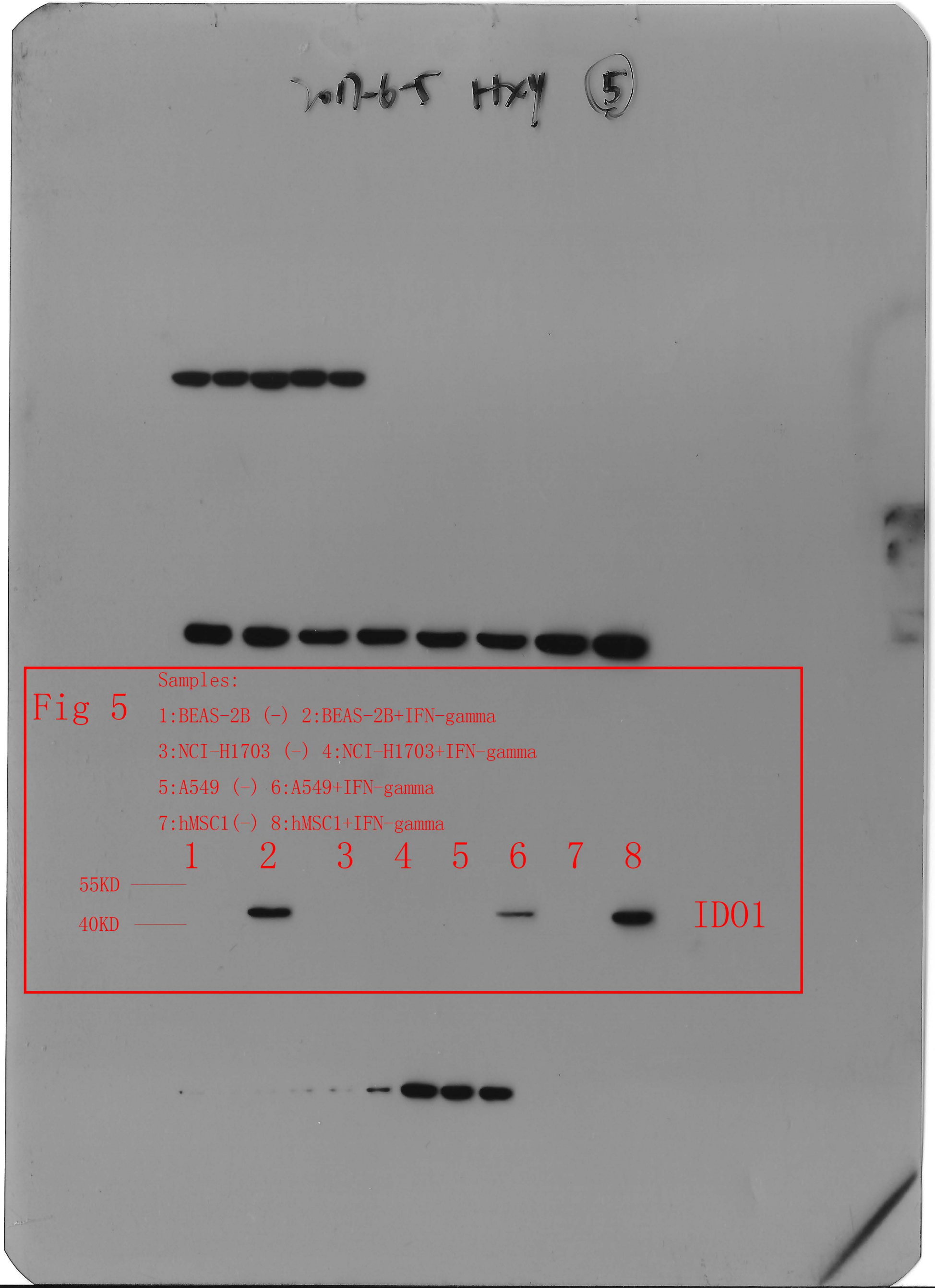
2017-6-5 Hxy 5
Fig 5
Samples:
1:BEAS-2B (-) 2:BEAS-2B+IFN-gamma
3:NCI-H1703 (-) 4:NCI-H1703+IFN-gamma
5:A549 (-) 6:A549+IFN-gamma
7:hMSC1(-) 8:hMSC1+IFN-gamma
1 2 3 4 5 6 7 8
55KD
40KD
IDO1

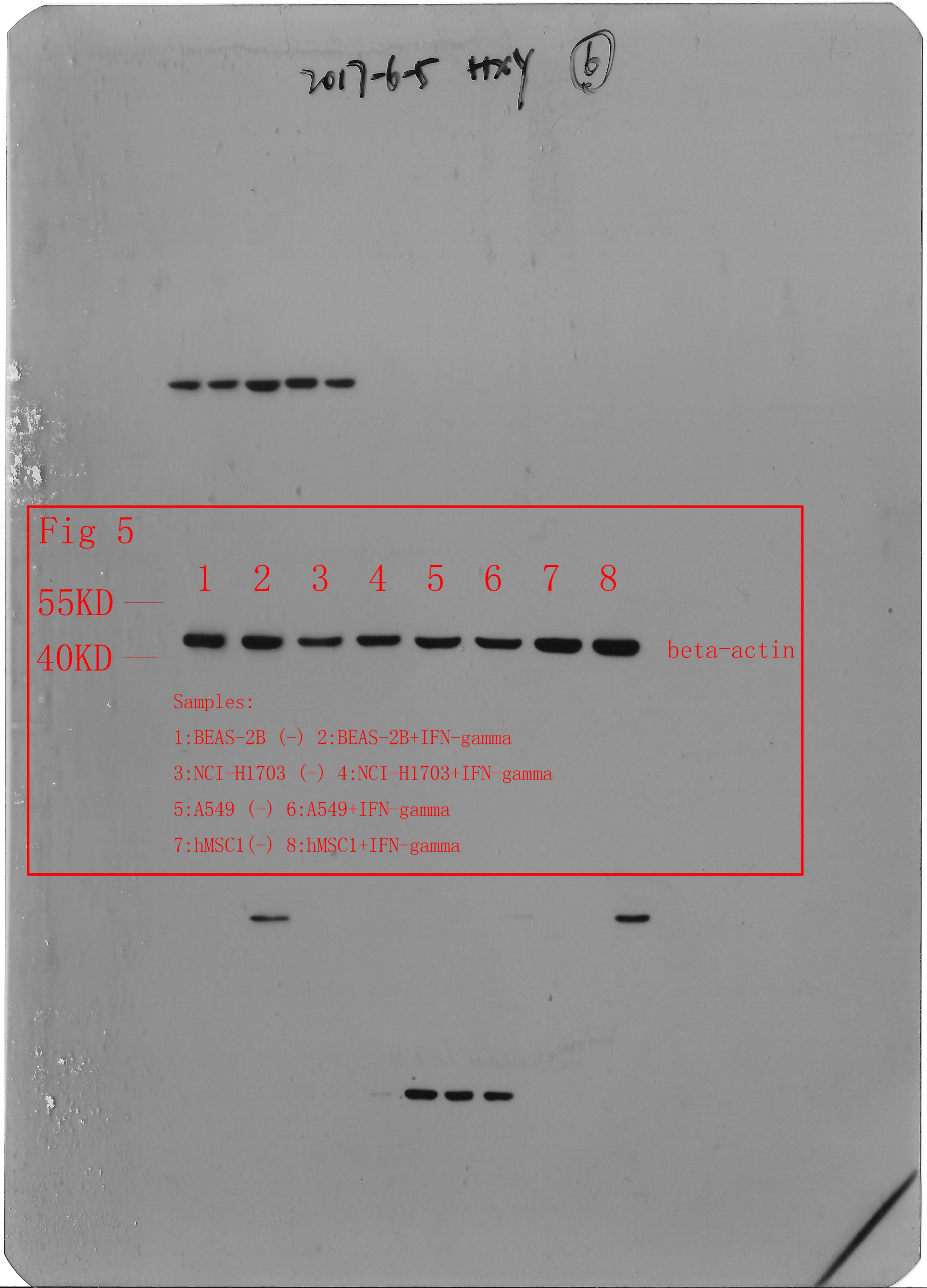
2017-6-5 Hxy 6
Fig 5
1 2 3 4 5 6 7 8
55KD
40KD
beta-actin
Samples:
1:BEAS-2B (-) 2:BEAS-2B+IFN-gamma
3:NCI-H1703 (-) 4:NCI-H1703+IFN-gamma
5:A549 (-) 6:A549+IFN-gamma
7:hMSC1(-) 8:hMSC1+IFN-gamma

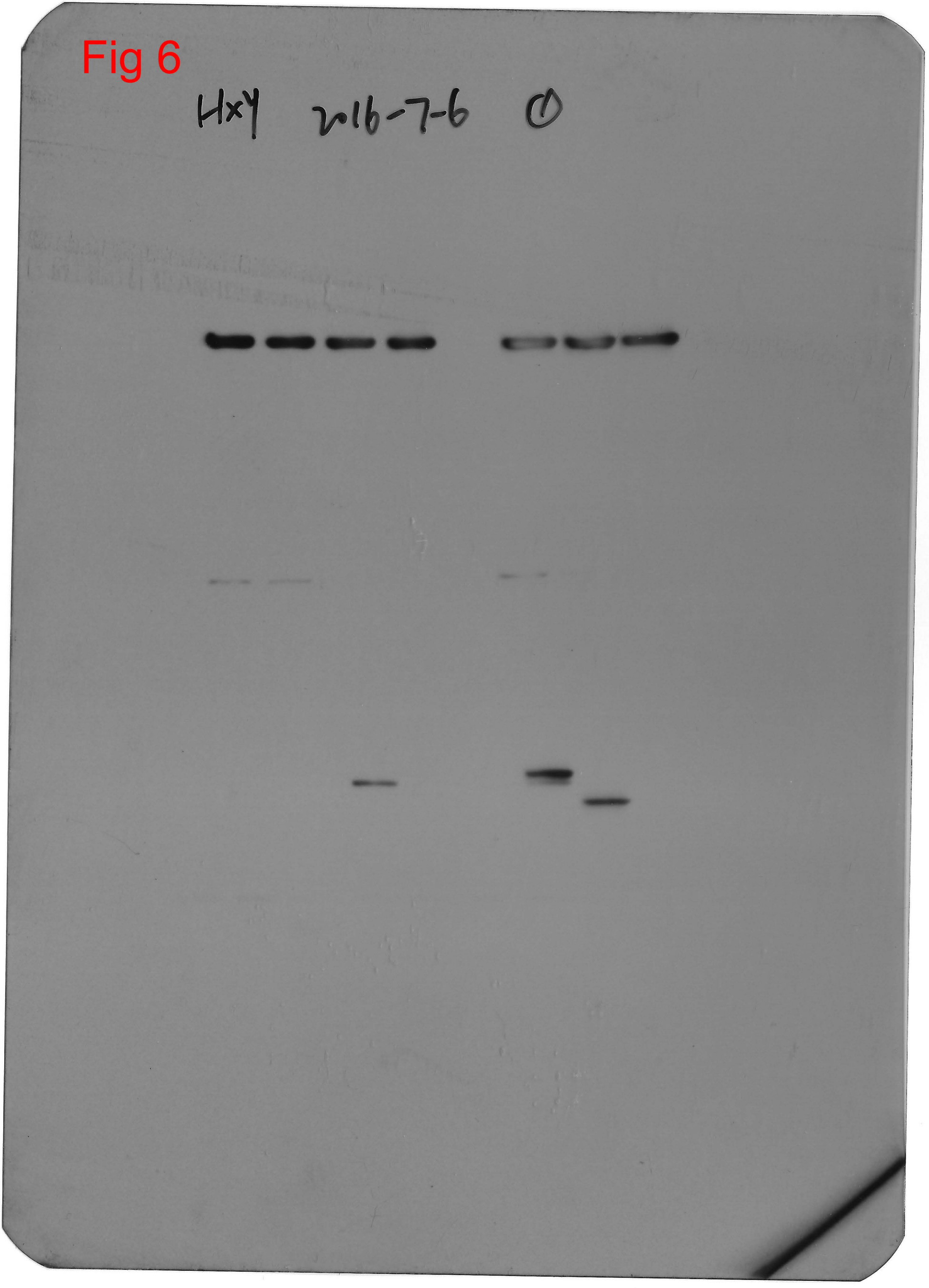
Fig 6
HxY 2016-7-6 ①

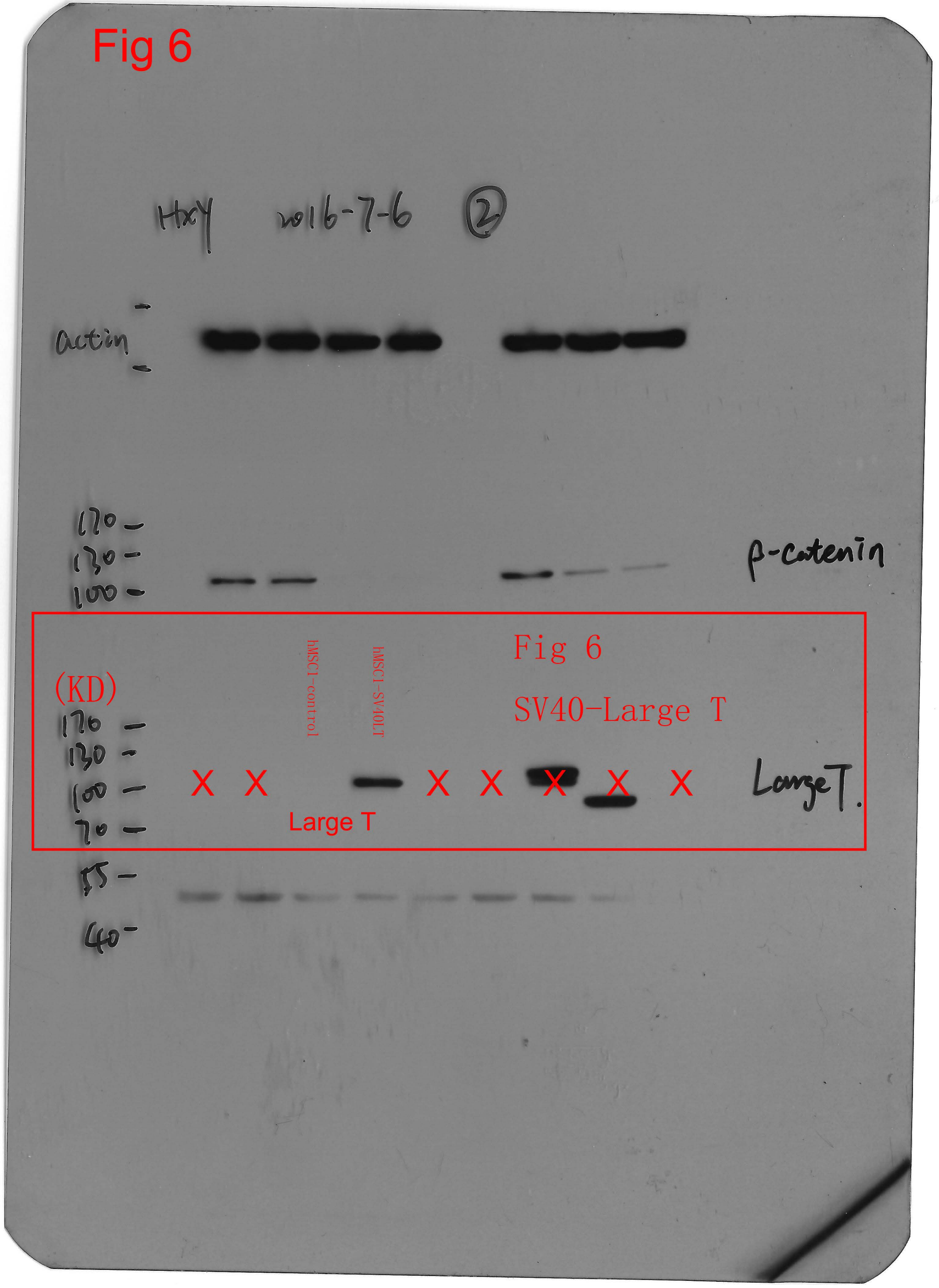
Fig 6
HxY 2016-7-6 ②
actin
170
130
100
β-catenin
(KD)
170
130
100
70
55
40
hMSC1-control
hMSC1-SV40LT
Fig 6
SV40-Large T
Large T
LargeT

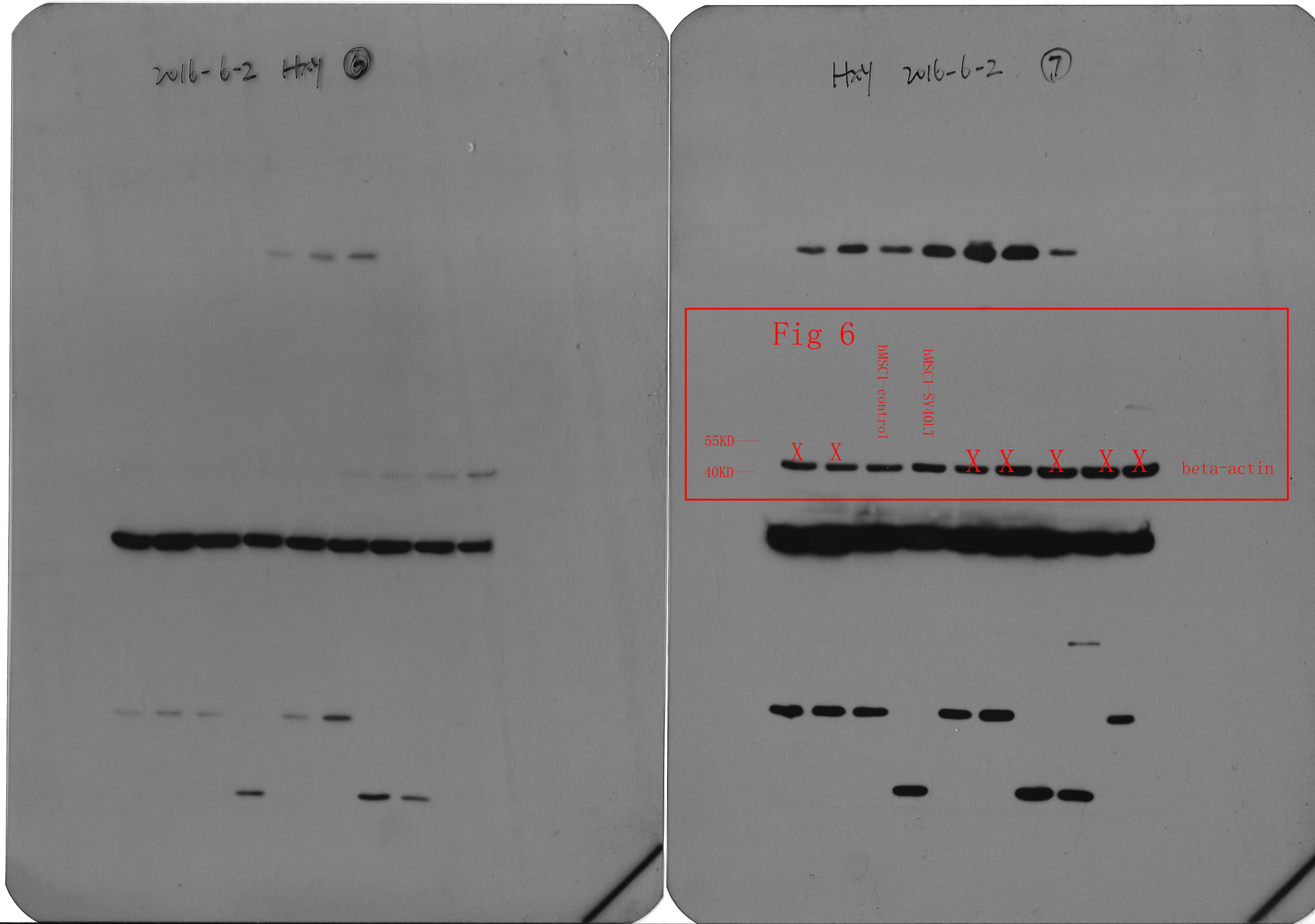
2016-6-2 Hxy 6
Hxy 2016-6-2 7
Fig 6
hMSC1-control
hMSC1-SV40LT
55KD
40KD
beta-actin

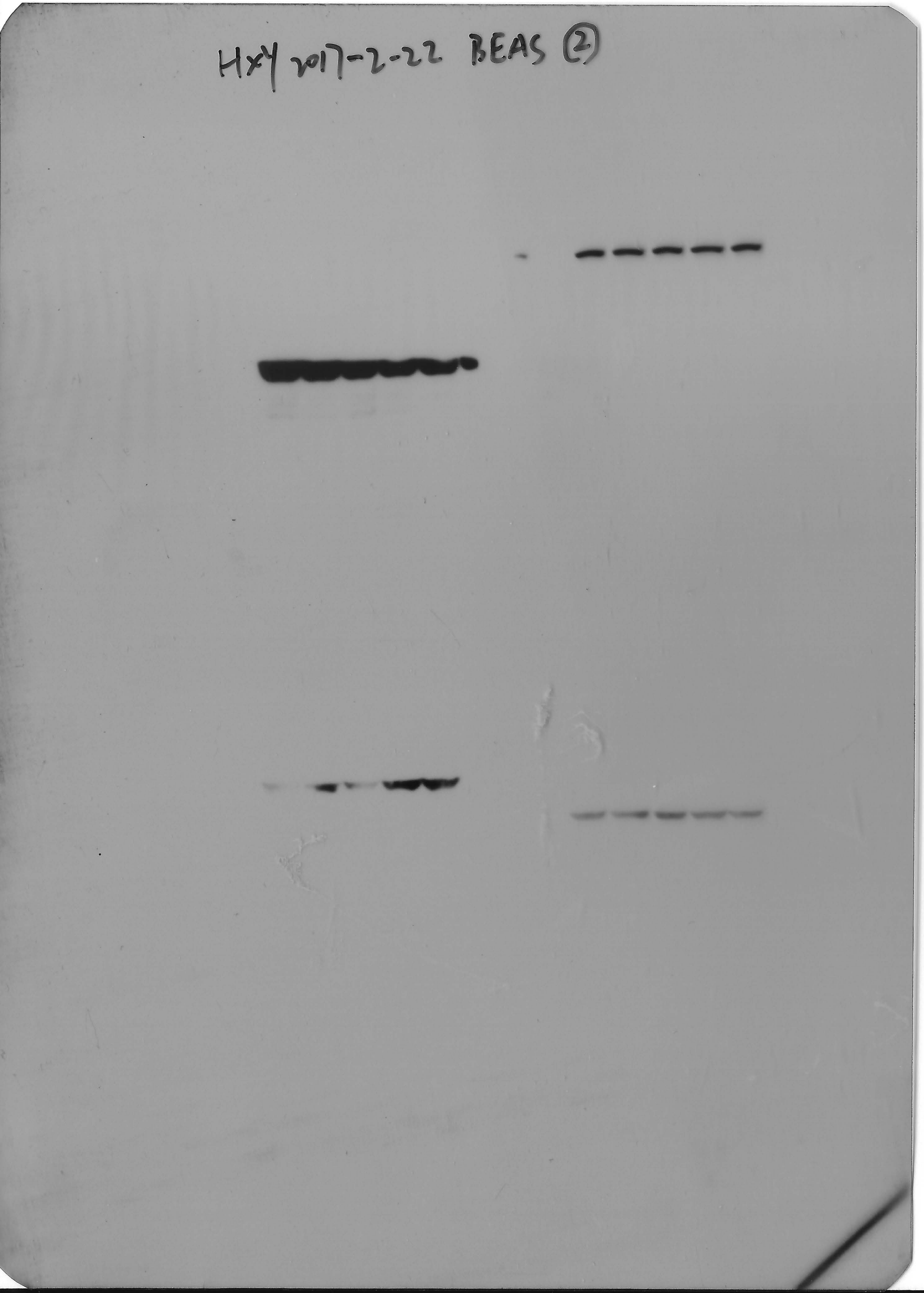
HXY 2017-2-22 BEAS ②

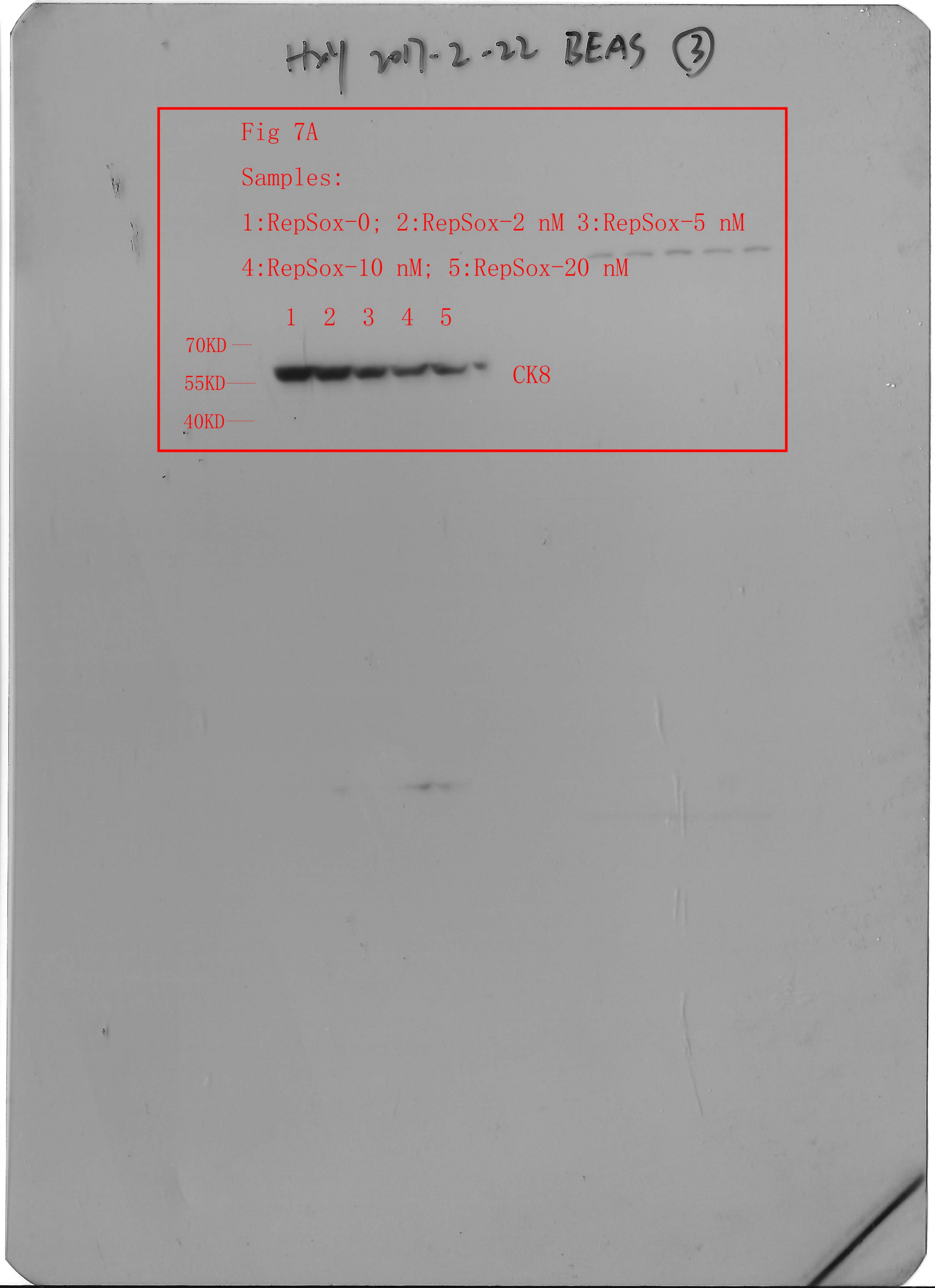
HXY 2017-2-22 BEAS ③
Fig 7A
Samples:
1:RepSox-0; 2:RepSox-2 nM 3:RepSox-5 nM
4:RepSox-10 nM; 5:RepSox-20 nM
1 2 3 4 5
70KD
55KD
40KD
CK8

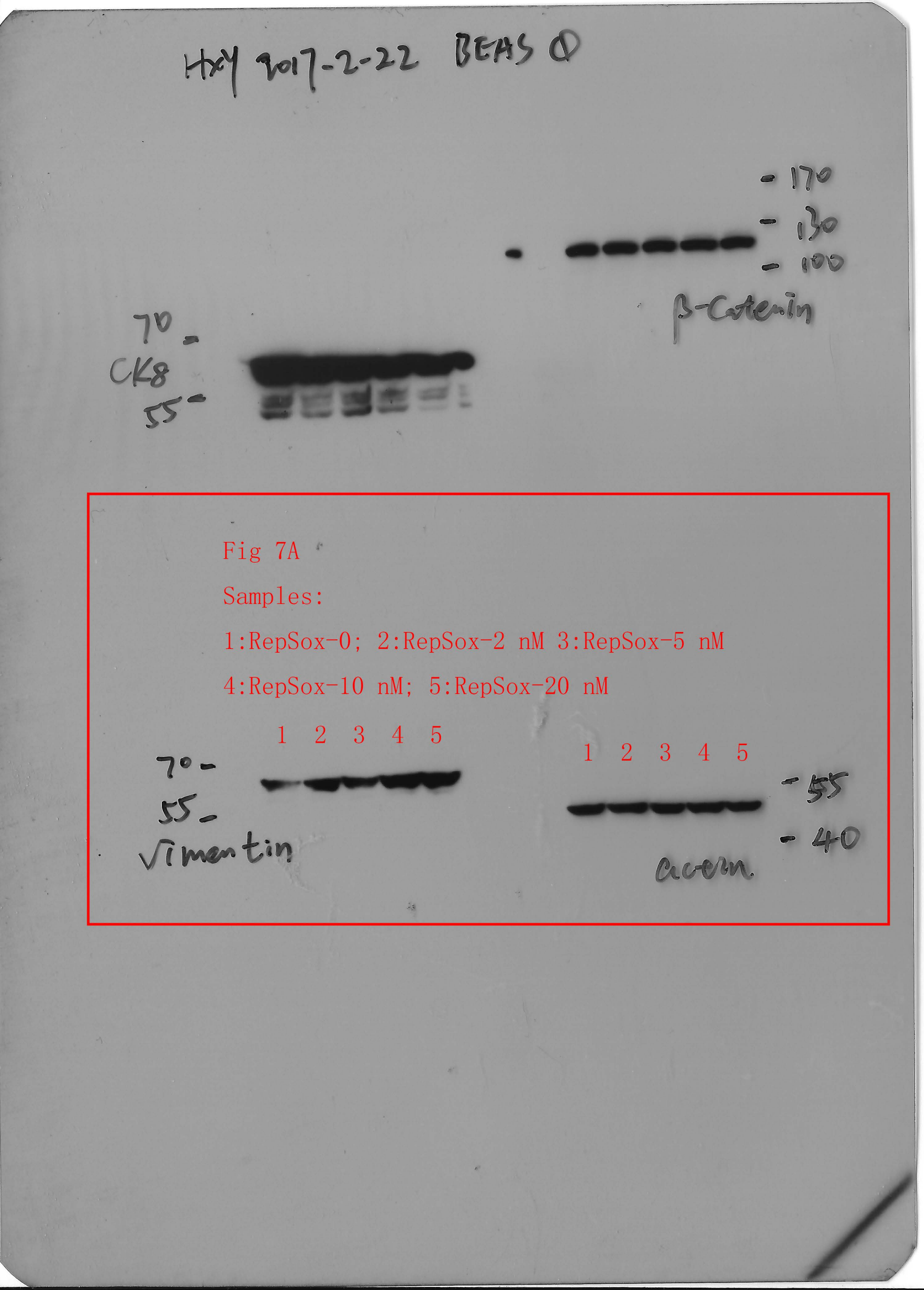
HXY 2017-2-22 BEAS ①
170
130
100
β-Catenin
70
CK8
55
Fig 7A
Samples:
1:RepSox-0; 2:RepSox-2 nM 3:RepSox-5 nM
4:RepSox-10 nM; 5:RepSox-20 nM
1 2 3 4 5
1 2 3 4 5
70
55
Vimentin
55
40
actin
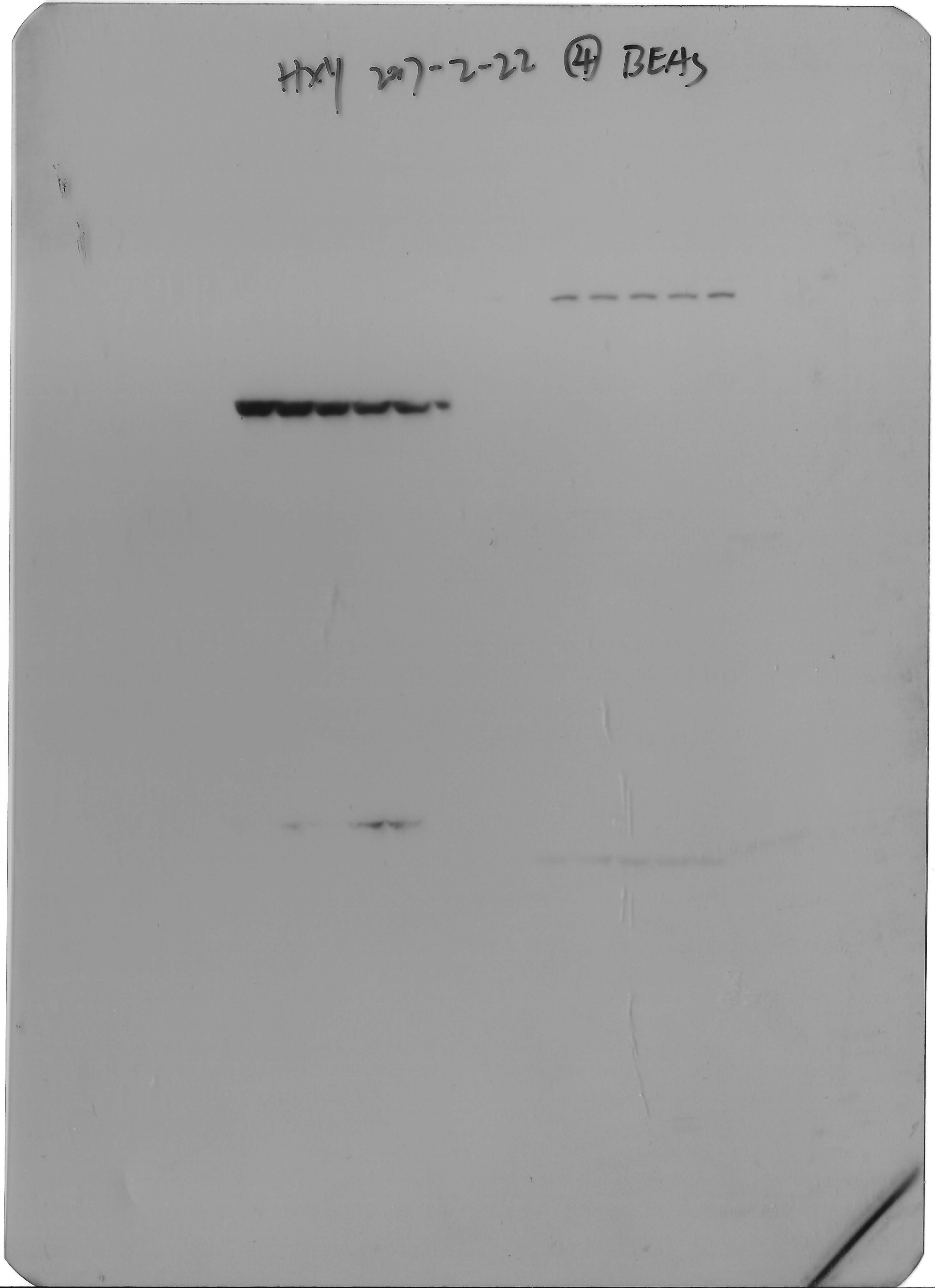
HXY 2017-2-22 ④ BEAS

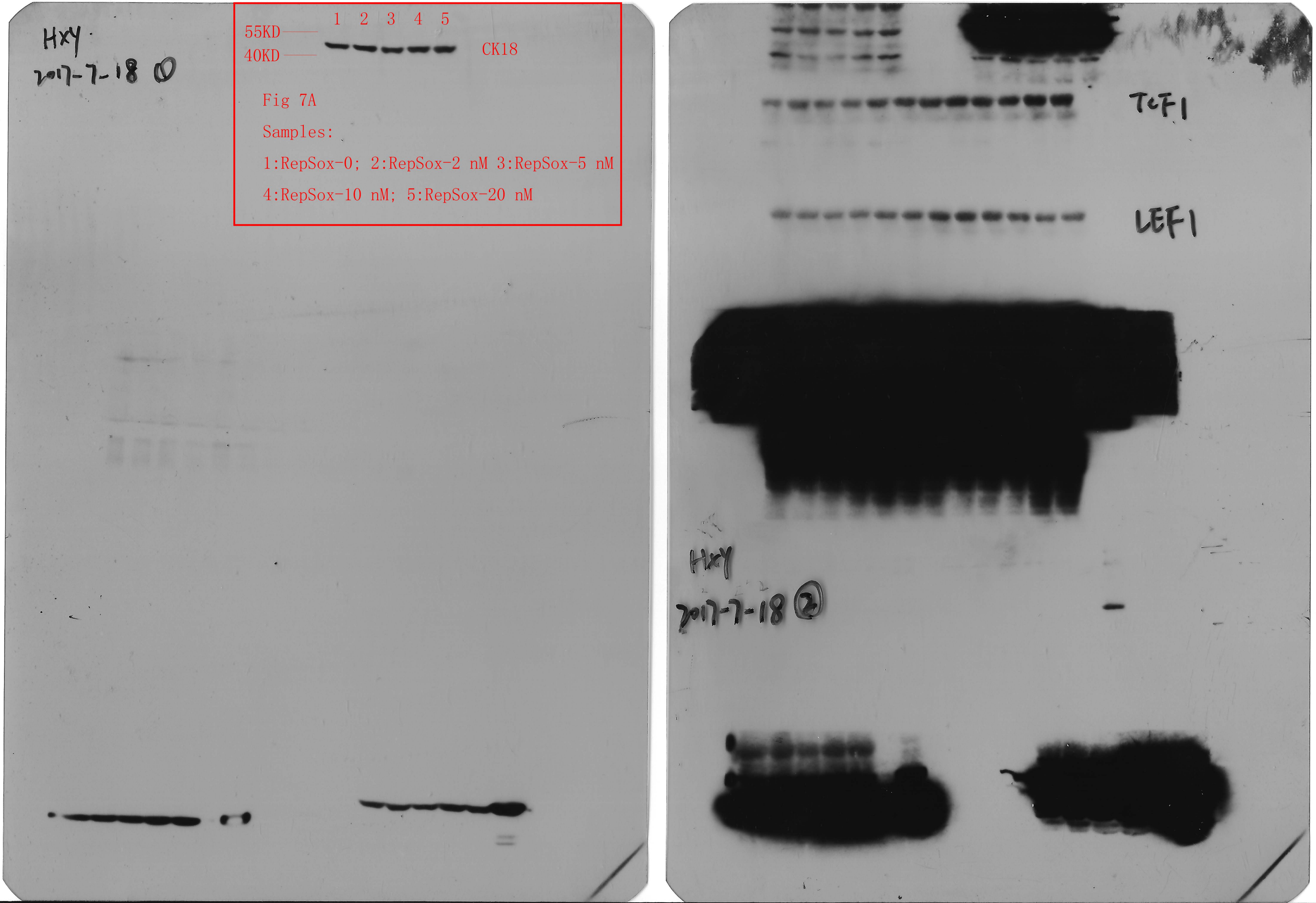
Hxy
2017-7-18 ①
1 2 3 4 5
55KD
40KD
CK18
Fig 7A
Samples:
1:RepSox-0; 2:RepSox-2 nM 3:RepSox-5 nM
4:RepSox-10 nM; 5:RepSox-20 nM
TCF1
LEF1
Hxy
2017-7-18 ②

Fig 7A

Samples:

1:RepSox-0; 2:RepSox-2 nM 3:RepSox-5 nM

4:RepSox-10 nM; 5:RepSox-20 nM

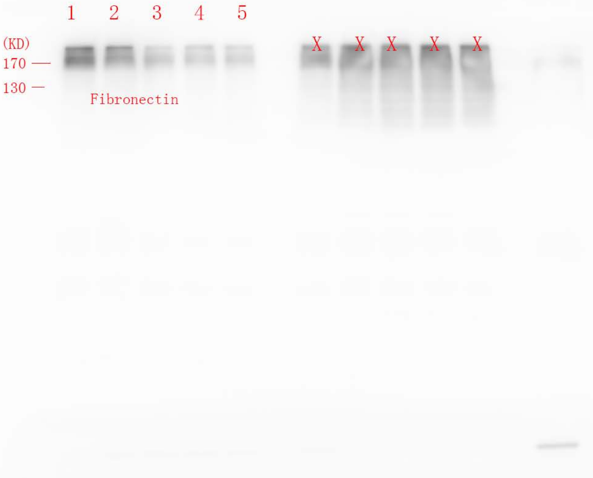

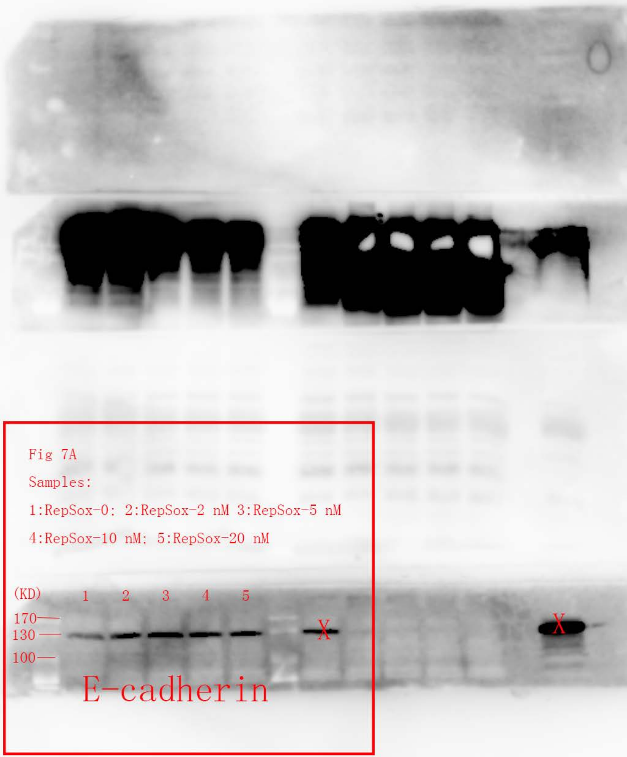
Fig 7A
Samples:
1:RepSox-0; 2:RepSox-2 nM 3:RepSox-5 nM
4:RepSox-10 nM; 5:RepSox-20 nM
(KD)
1
2
3
4
5
170
130
100
X
X
E-cadherin

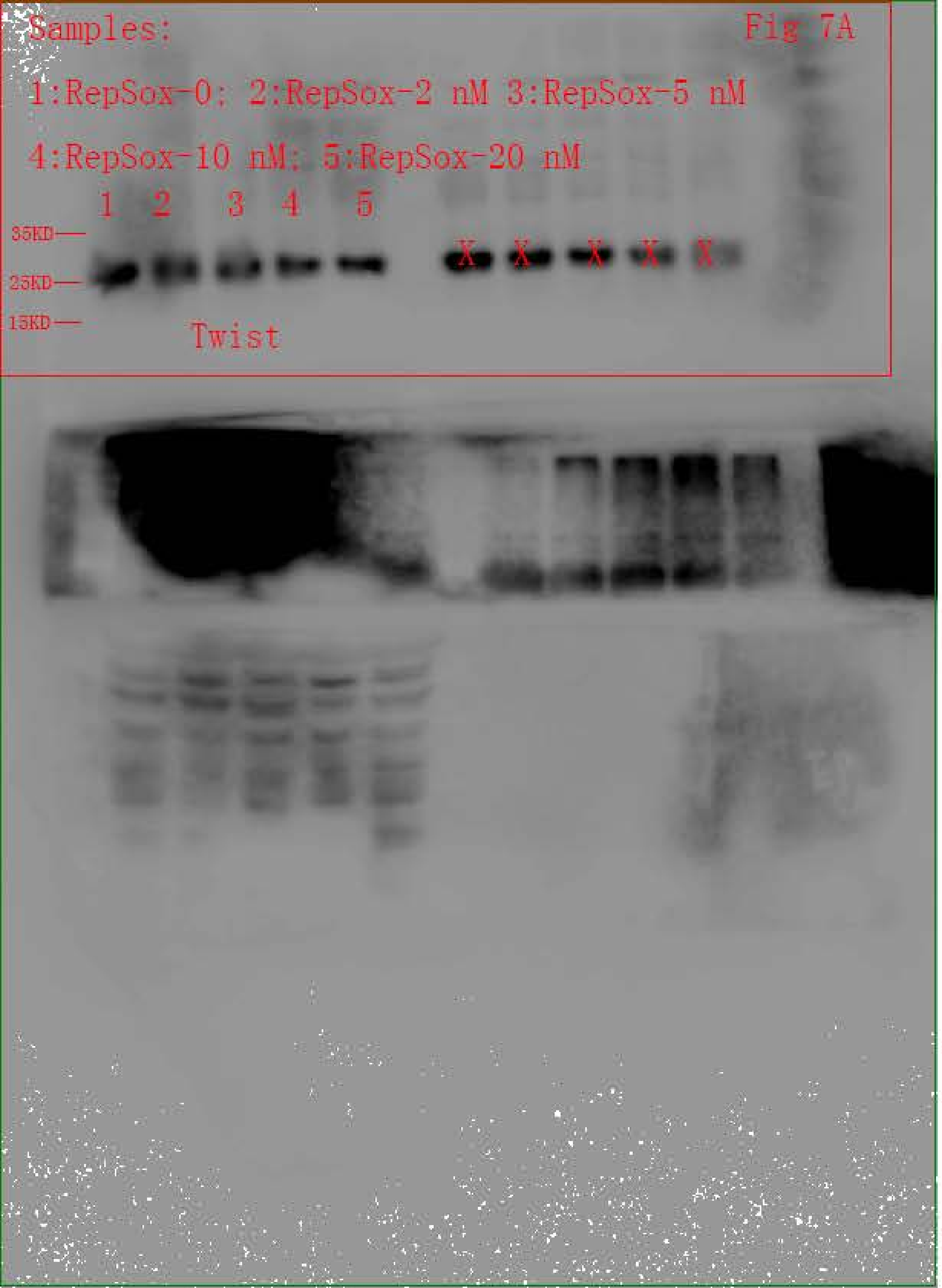
Samples:
Fig 7A
1:RepSox-0; 2:RepSox-2 nM 3:RepSox-5 nM
4:RepSox-10 nM; 5:RepSox-20 nM
1 2 3 4 5
35KD
25KD
15KD
Twist

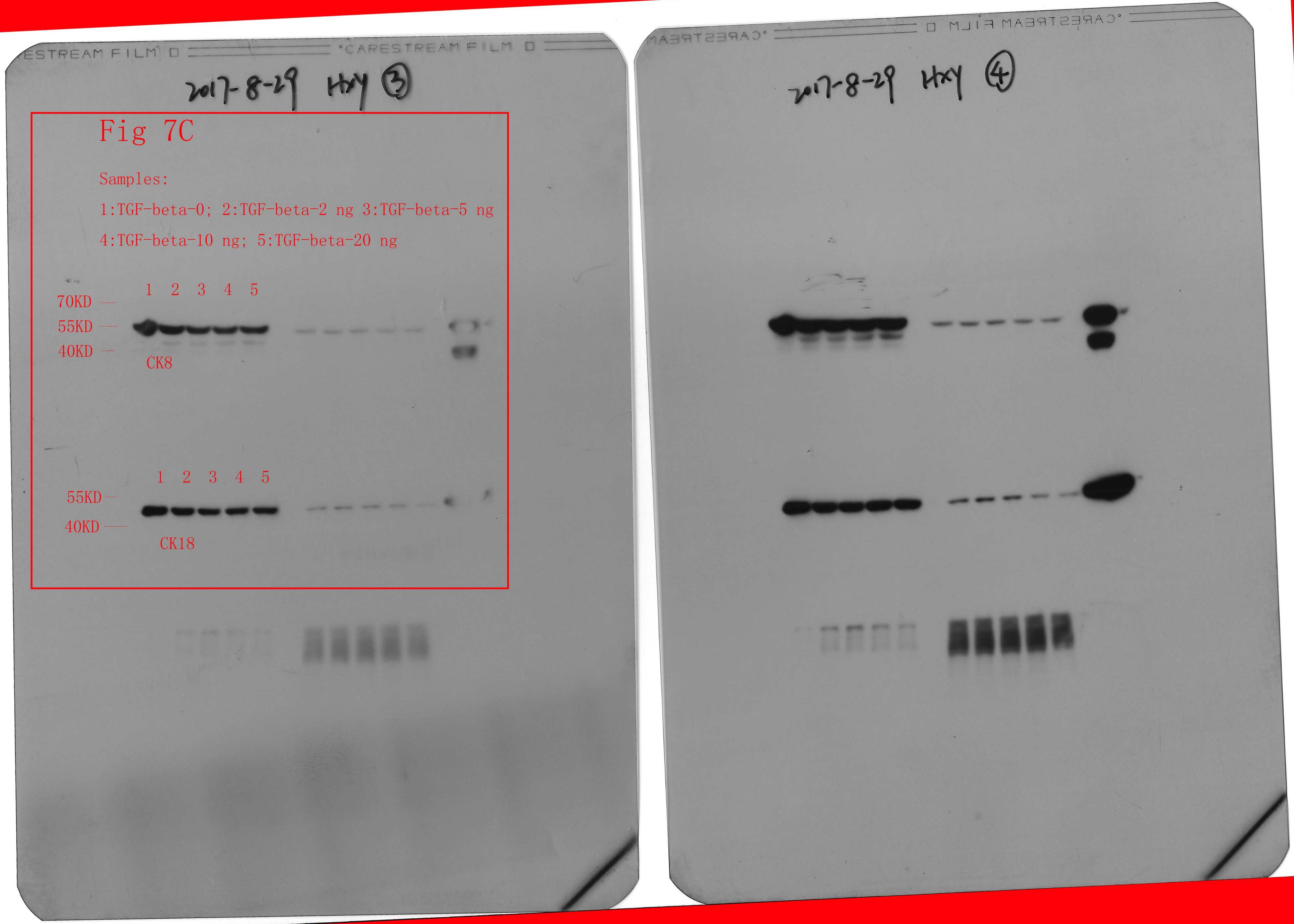
CARESTREAM FILM
2017-8-29 Hxy ③
Fig 7C
Samples:
1:TGF-beta-0; 2:TGF-beta-2 ng 3:TGF-beta-5 ng
4:TGF-beta-10 ng; 5:TGF-beta-20 ng
1 2 3 4 5
70KD
55KD
40KD
CK8
1 2 3 4 5
55KD
40KD
CK18
2017-8-29 Hxy ④

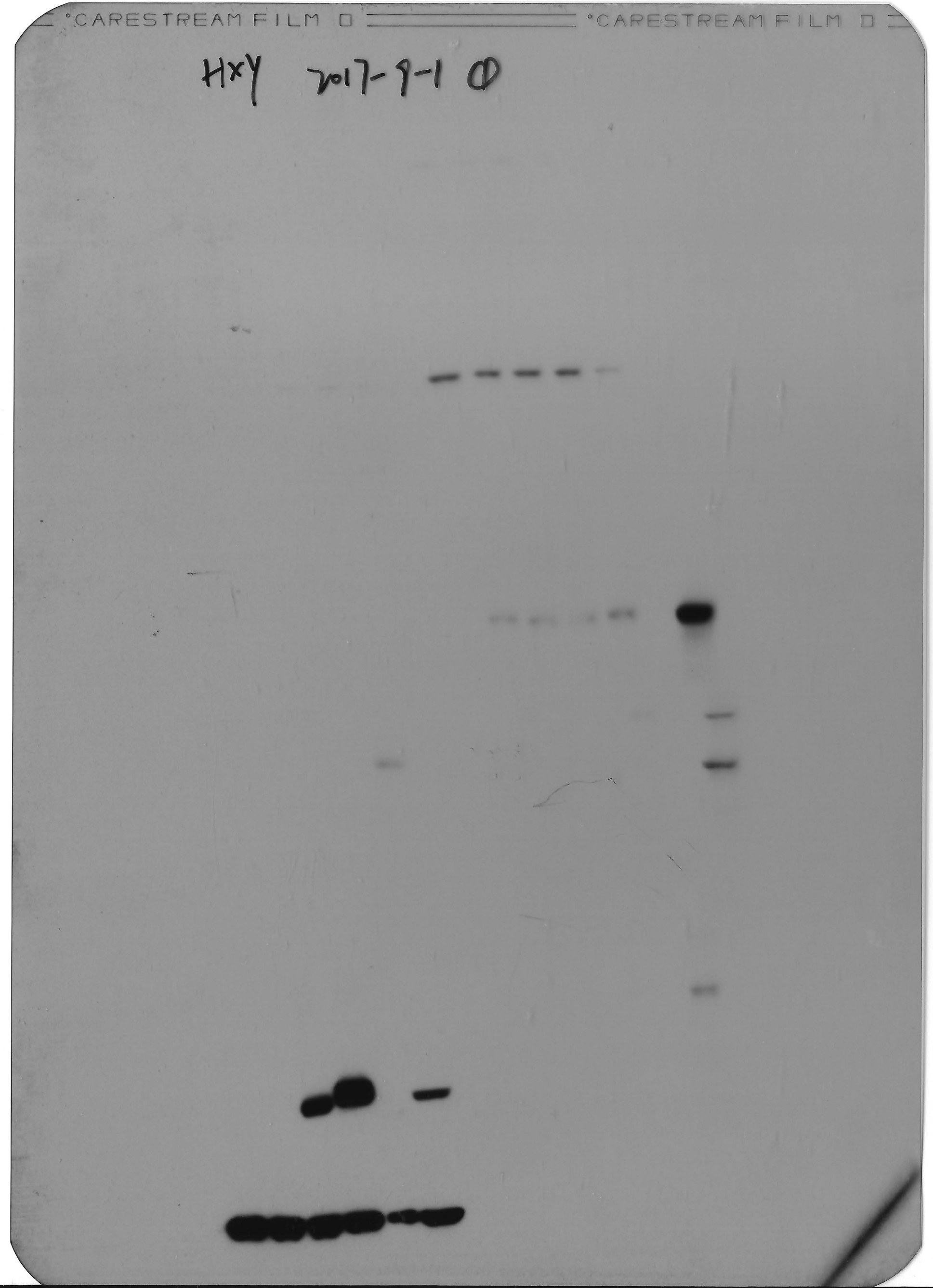
CARESTREAM FILM
CARESTREAM FILM
Hxy 2017-9-1 ①

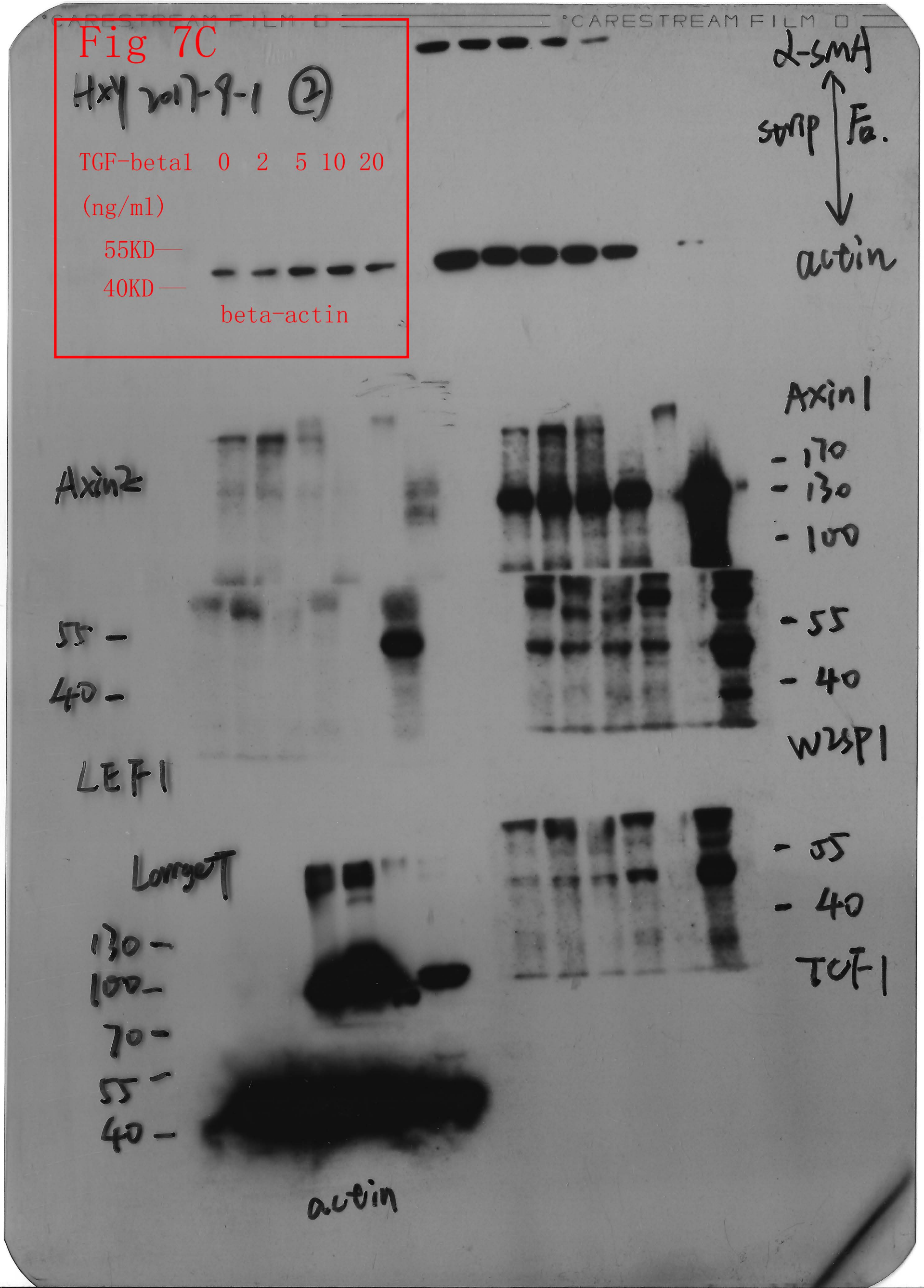
CARESTREAM FILM
Fig 7C
Hxy 2017-9-1 ②
TGF-beta1 0 2 5 10 20
(ng/ml)
55KD
40KD
beta-actin
α-SMA
strip
Fa.
actin
Axin1
- 170
- 130
- 100
- 55
- 40
Axin2
55 -
40 -
LEF1
LargeT
130 -
100 -
70 -
55 -
40 -
actin
- 55
- 40
TCF1

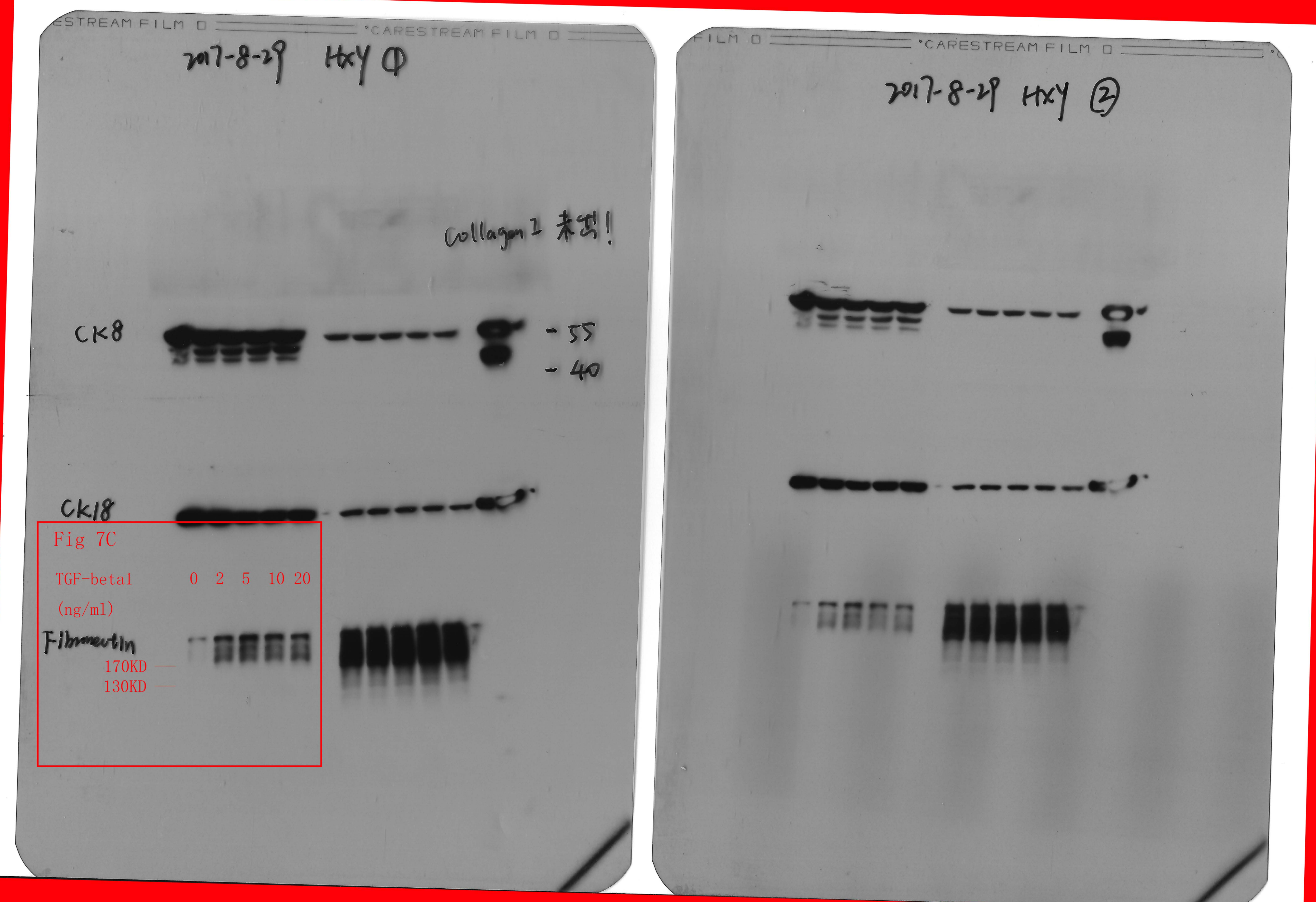
CARESTREAM FILM
2017-8-29 HxY ①
Collagen I 未出!
CK8
- 55
- 40
Ck18
Fig 7C
TGF-beta1
0 2 5 10 20
(ng/ml)
Fibronectin
170KD
130KD
2017-8-29 HxY ②

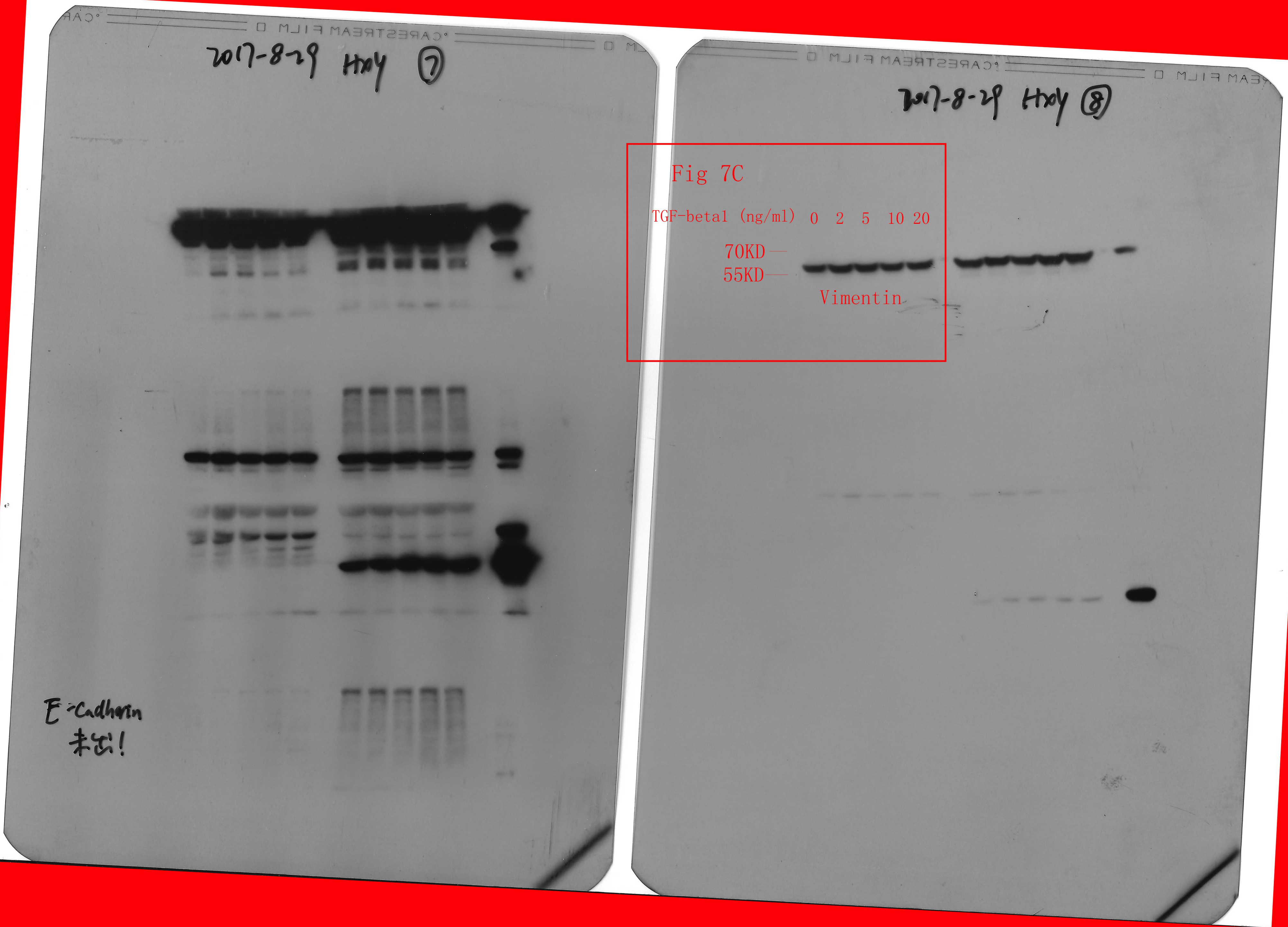
2017-8-29 Hxy ⑦
2017-8-29 Hxy ⑧
Fig 7C
TGF-beta1 (ng/ml) 0 2 5 10 20
70KD
55KD
Vimentin
E-Cadherin
未出!

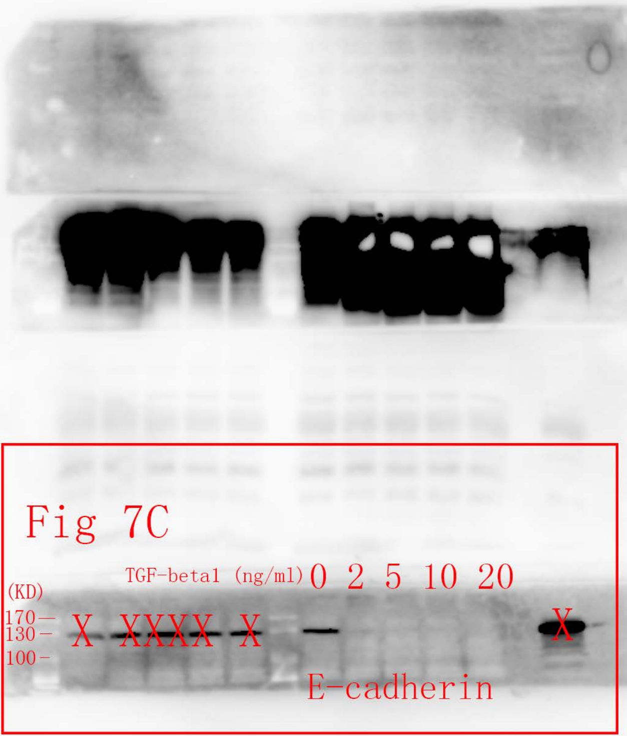
Fig 7C
TGF-beta1 (ng/ml) 0 2 5 10 20
(KD)
170
130
100
E-cadherin

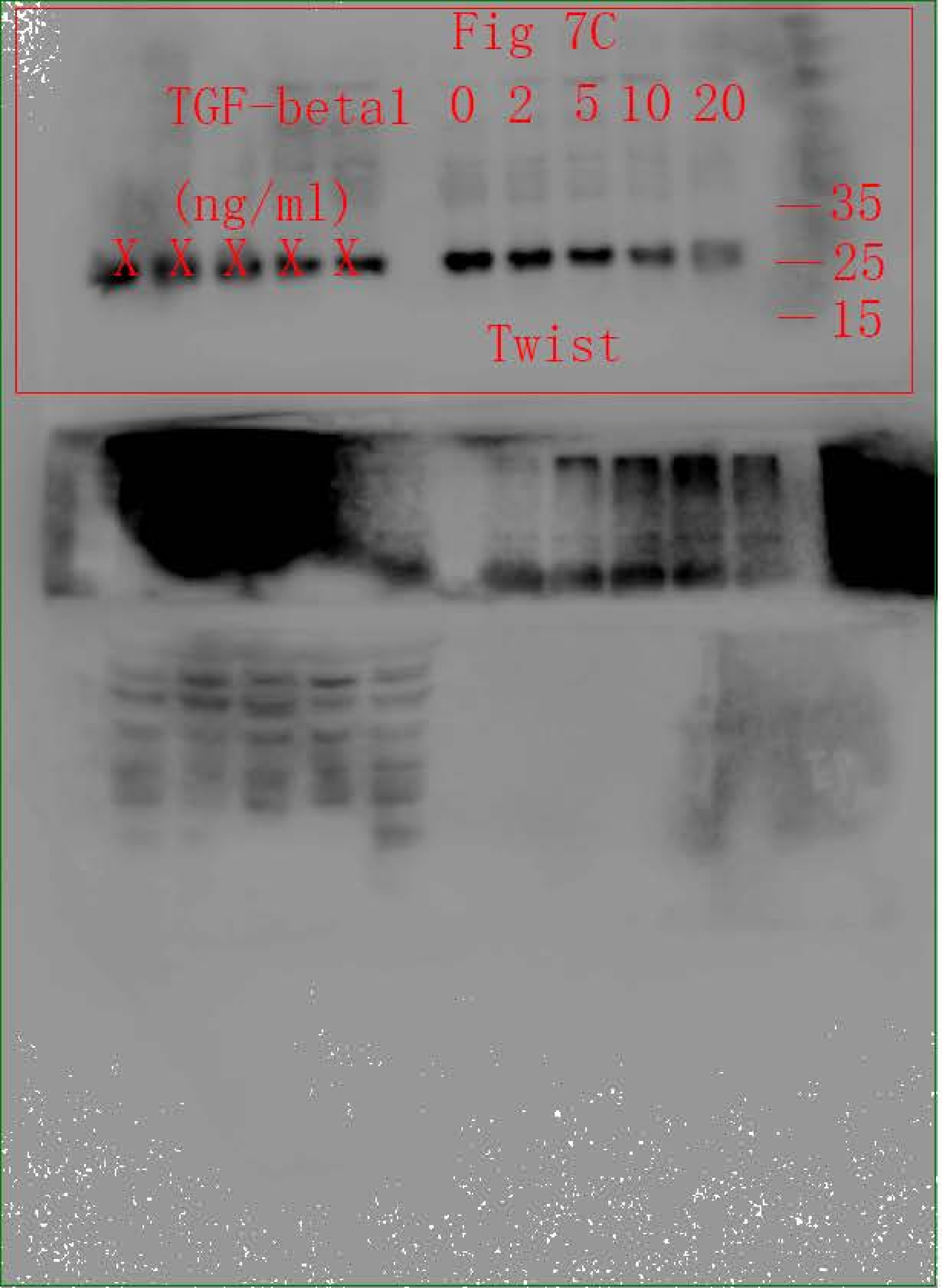
Fig 7C
TGF-beta1 0 2 5 10 20
(ng/ml)
X X X X X
35
25
15
Twist

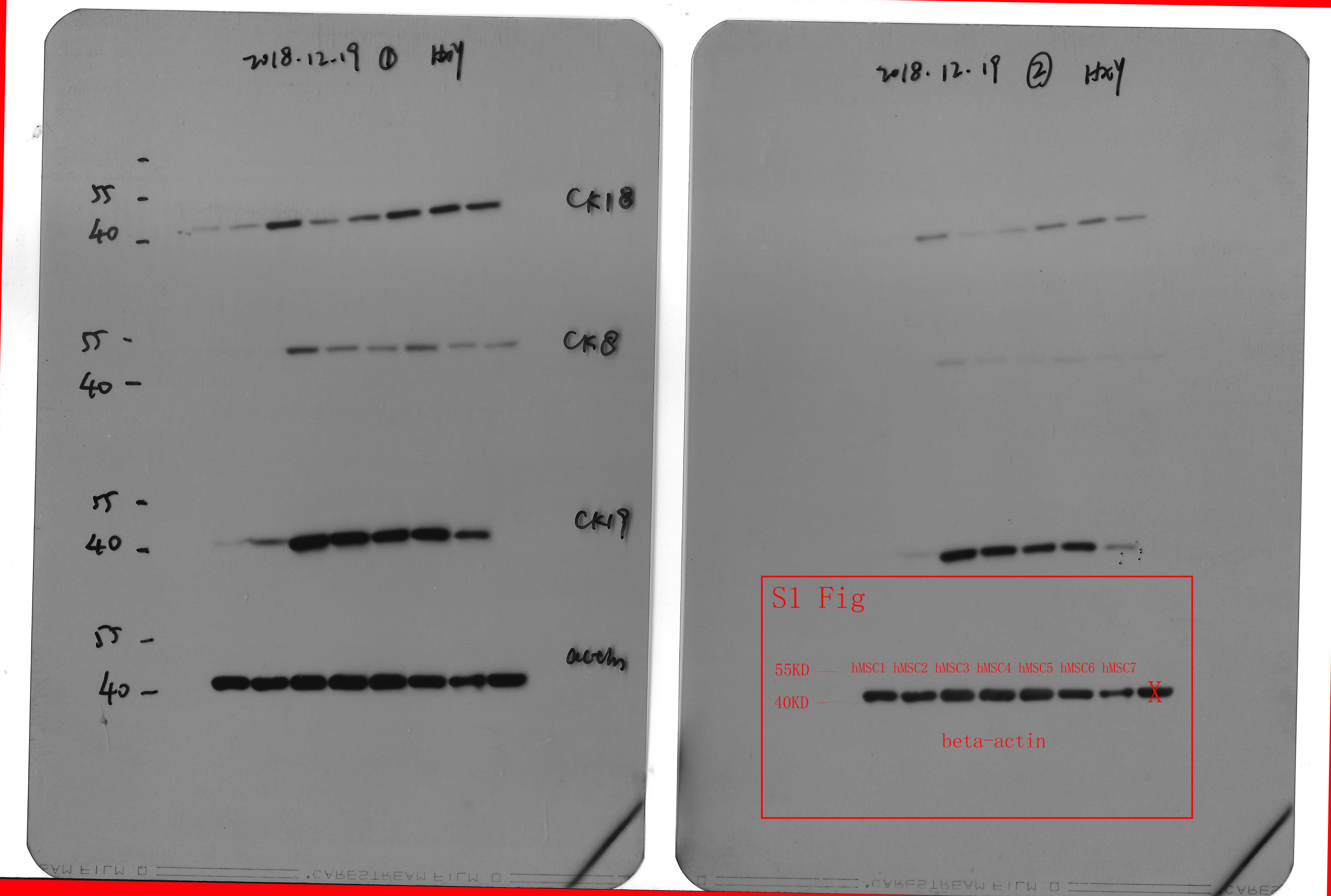
2018.12.19 ① Hxy
55
40
CK18
CK8
CK19
actin
2018.12.19 ② Hxy
S1 Fig
55KD
40KD
hMSC1 hMSC2 hMSC3 hMSC4 hMSC5 hMSC6 hMSC7
X
beta-actin

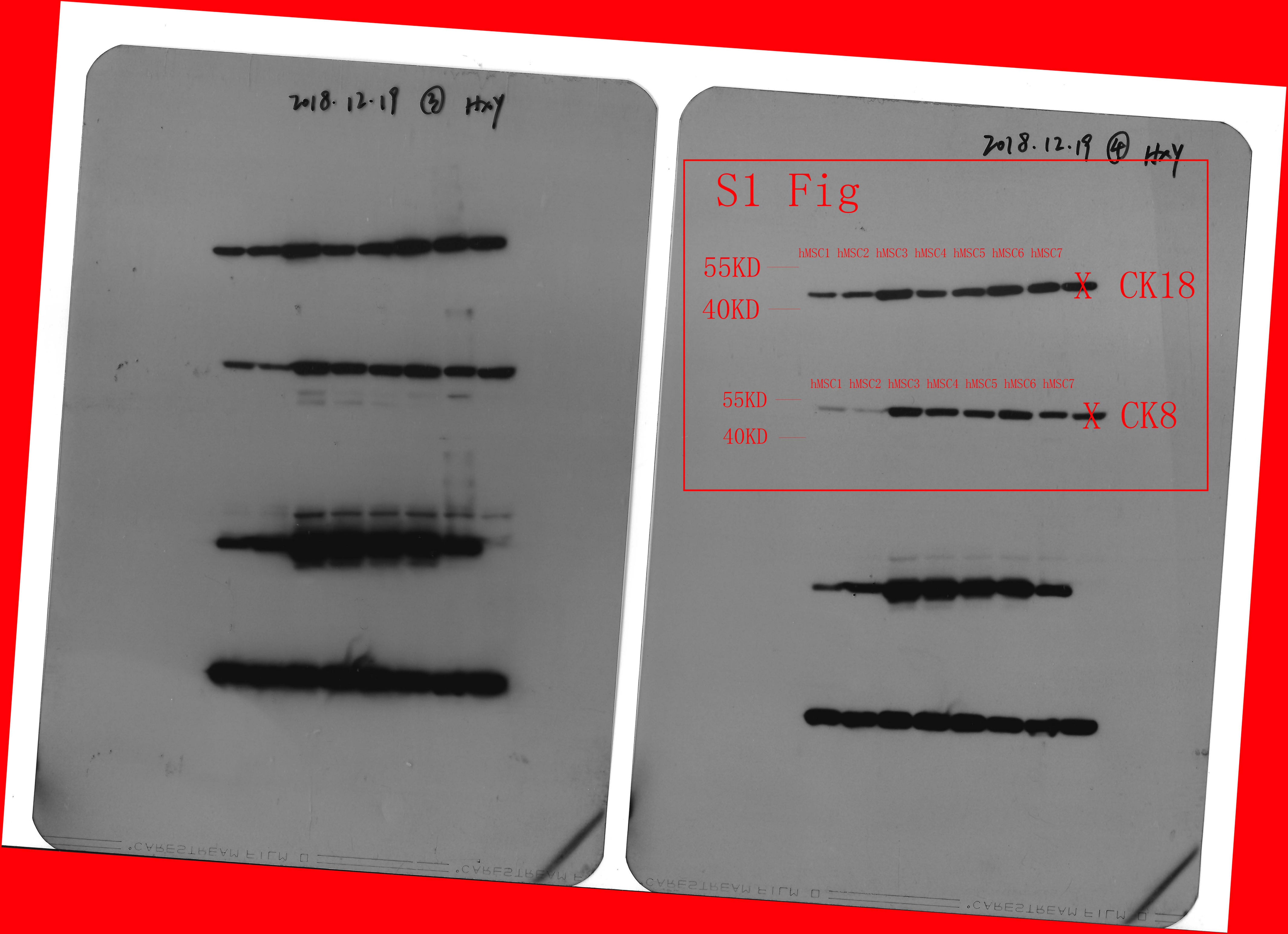
2018.12.19 ③ HxY
2018.12.19 ④ HxY
S1 Fig
hMSC1 hMSC2 hMSC3 hMSC4 hMSC5 hMSC6 hMSC7
55KD
40KD
X CK18
hMSC1 hMSC2 hMSC3 hMSC4 hMSC5 hMSC6 hMSC7
55KD
40KD
X CK8
CARESTREAM FILM

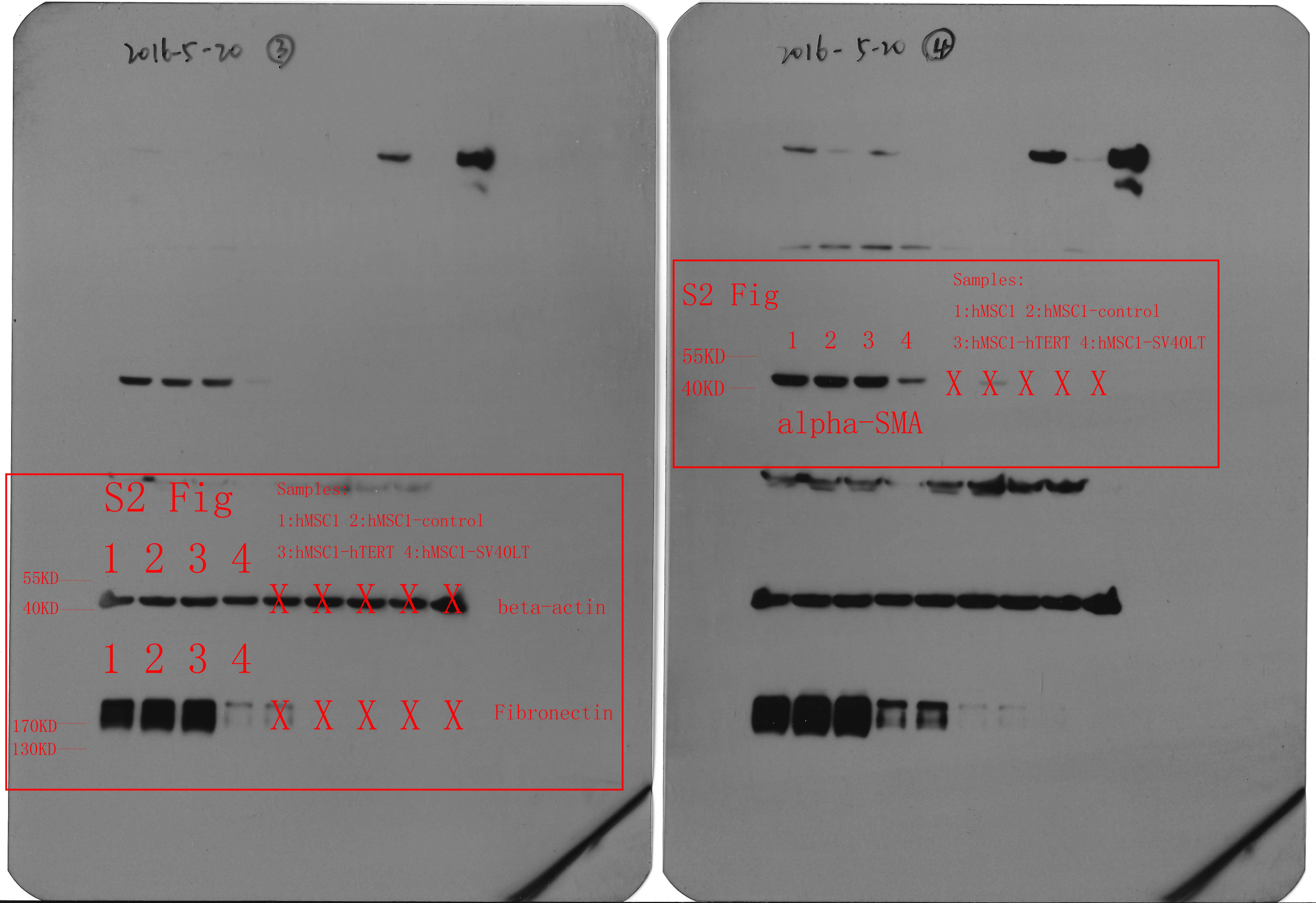

2016-5-20 ③
2016-5-20 ④
S2 Fig
Samples:
1:hMSC1 2:hMSC1-control
3:hMSC1-hTERT 4:hMSC1-SV40LT
1 2 3 4
55KD
40KD
beta-actin
1 2 3 4
170KD
130KD
Fibronectin
S2 Fig
Samples:
1:hMSC1 2:hMSC1-control
3:hMSC1-hTERT 4:hMSC1-SV40LT
1 2 3 4
55KD
40KD
alpha-SMA

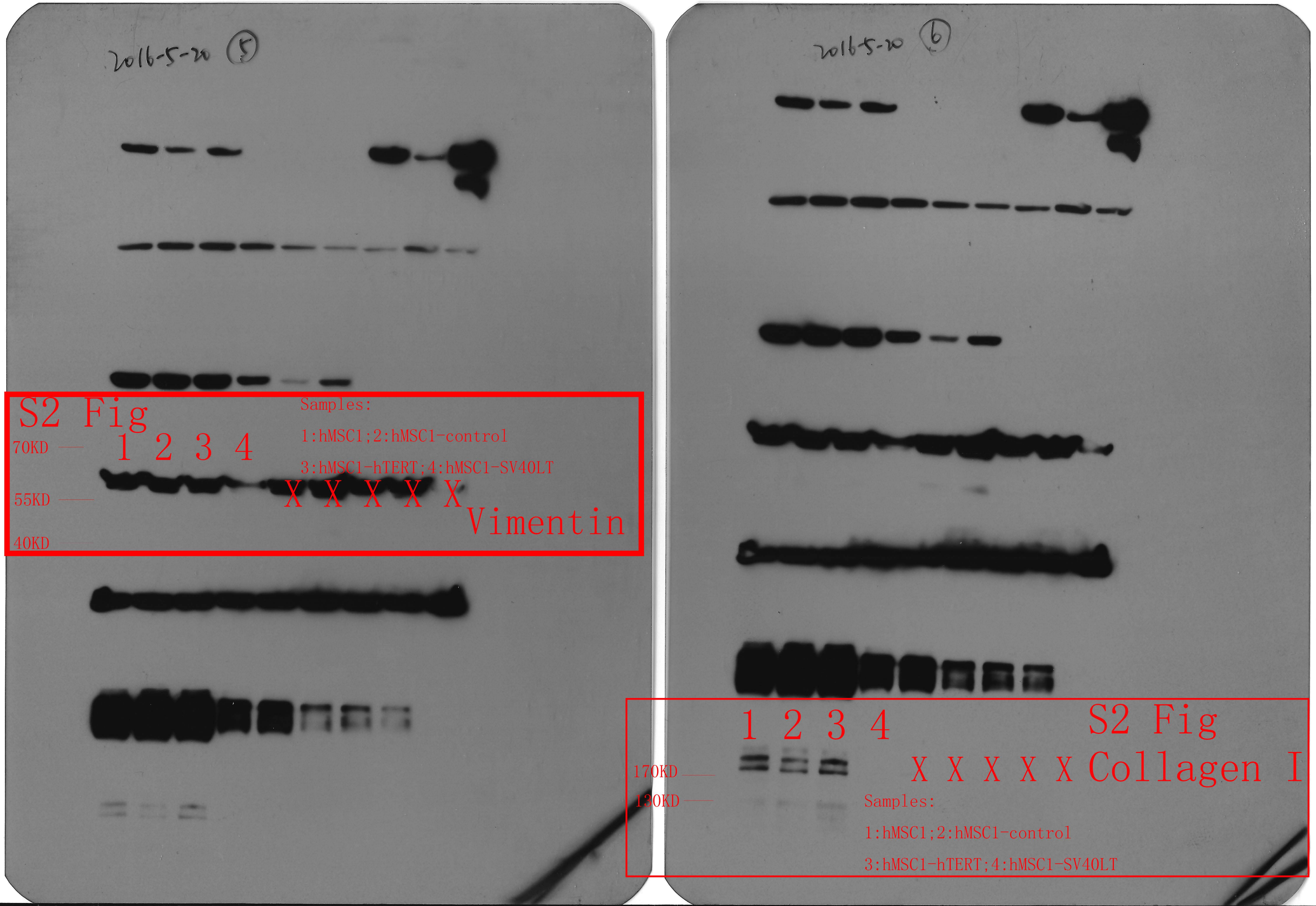
2016-5-20 ⑤
S2 Fig
1 2 3 4
70KD
55KD
40KD
X X X X X
Vimentin
Samples:
1:hMSC1;2:hMSC1-control
3:hMSC1-hTERT;4:hMSC1-SV40LT
2016-5-20 ⑥
1 2 3 4
S2 Fig
X X X X X
Collagen I
170KD
130KD
Samples:
1:hMSC1;2:hMSC1-control
3:hMSC1-hTERT;4:hMSC1-SV40LT
